# In their own words: case studies of adolescent smartphone language preceding suicide-related hospitalizations

**DOI:** 10.1038/s44277-026-00057-0

**Published:** 2026-03-02

**Authors:** Isaac N. Treves, Paul A. Bloom, Samantha Salem, Katherine Durham, Valerio Zaccaria, Jamaal Spence, Peter S. Dayan, Lauren S. Chernick, Ashley Blanchard, Jaclyn S. Kirshenbaum, Esha Trivedi, David A. Brent, Nicholas B. Allen, Jamie Zelazny, Karla Joyce, Giovanna Porta, David Pagliaccio, Randy P. Auerbach

**Affiliations:** 1 Department of Psychiatry, Columbia University, New York, US; 2 Department of Human Neuroscience, Sapienza University of Rome, Rome, Italy; 3 Department of Emergency Medicine, Columbia University, New York, US; 4 Division of Child and Adolescent Psychiatry, New York State Psychiatric Institute, New York, US; 5 Department of Psychiatry, University of Pittsburgh Medical Center, Pittsburgh, US; 6 Department of Psychology, University of Oregon, Eugene, US; 7 School of Nursing, University of Pittsburgh, Pittsburgh, US; 8 Western Psychiatric Hospital, University of Pittsburgh Medical Center, Pittsburgh, US

**Keywords:** Risk factors, Paediatric research, Human behaviour

## Abstract

Rising adolescent suicide rates underscore an urgent need for better detection of short-term risk in the days and hours leading up to attempts. Passive smartphone sensing of language offers a promising approach, yet its performance during vulnerable periods remains unclear. This case study examined five adolescents (3 male, 2 female) who were hospitalized for suicidal crises while enrolled in a smartphone sensing study. Participants contributed outgoing text entries over six months (M = 21,000/person), which were analyzed using natural language processing (NLP) to assess suicide-related content, sentiment, and topics (e.g., school, treatment). In addition, clinicians conducted qualitative reviews of the text entries to identify potential risk events. Results showed that 4 of 5 adolescents exhibited increased suicide-related language and negative sentiment during the 10 days prior to psychiatric hospitalization. Especially elevated suicide language was found within 5 days of hospitalization, while negative sentiment peaked between 5–10 days prior to hospitalization. These signals, however, also occurred outside of acute risk periods, highlighting the challenge of separating suicide risk from distress more generally. Clinical annotations revealed that suicidal thoughts and behaviors often co-occurred with NLP signals of suicide-related language, and topic models identified clinically relevant language related to substance use and psychiatric treatment. Clinical annotations of interpersonal conflict and school stressors were not identified by topic models. Discrepancies largely originated from the inability of NLP methods to infer context (e.g., text conversation history). Although smartphone language data showed low missingness and some sensitivity to acute crises, enhancing contextual analysis is essential for personalized risk detection.

## Lay Summary

Smartphone communication in adolescents could reveal emerging emotions and behaviors that predict suicide risk. It is unclear how effective computational methods including AI could be for identifying risk. In the current study, smartphone keyboard inputs were collected for weeks prior to suicide hospitalization in five adolescents. Findings demonstrated consistent elevations in suicidal language and negative sentiment in the immediate period before hospitalization. Clinicians noted additional language (e.g., interpersonal conflicts) not captured by computational methods.

## Introduction

Suicide is a leading cause of death among youth in the United States [1, 2], with an increasing prevalence in recent years. Research has traditionally focused on identifying *who* is at risk, however, emerging perspectives argue for a shift in emphasis toward detecting *when* individuals are at risk for suicidal thoughts and behaviors (STB) [3–6]^,^ This reframing has been supported by findings from smartphone ecological momentary assessment (EMA) studies, which have demonstrated through frequent assessments that suicidal thoughts and related risk factors fluctuate significantly within short timeframes [7, 8], even within a single day [9, 10]. Robustly detecting these fluctuations in risk could allow for more effective treatment and prevention.

In recent years, passive sensing technologies, which collect data unobtrusively from personal digital devices (e.g., smartphones, wearables), have emerged as powerful tools for capturing psychological and behavioral states in real time, offering a promising complement to self-reported EMA [11–13]. These methods can detect patterns in mobility [14, 15], keyboard usage [16], sleep [17], physical activity [18], physiology [19], and social interactions [20], providing rich, continuous indicators of daily functioning. As passive sensing may offer higher temporal resolution and pose less burden to participants than responding to EMA surveys, digital phenotyping methods have particular promise for studying the temporal dynamics of STB [21, 22].

Among passive sensing modalities, smartphone-typed text offers a particularly rich window into adolescents’ real-time thoughts, emotions, and behaviors. Text can provide insights beyond what may be disclosed by youth in surveys or interviews, and reflects day-to-day communication with peers and family across various apps, as well as non-interpersonal entries, such as web searches or notes [23, 24]. Recent work has found that natural language processing (NLP) can uncover linguistic signals indicative of mental health symptoms [25–28]. For example, the use of increased negative sentiment or self-referential language (e.g., first-person pronouns) can presage negative mood and depressive episodes in youth [29, 30], while typed language reflecting suicidal ideation has predicted elevated suicide risk in adults [31]. Recent work also indicates suicide-relevant language can be detected via youth smartphone keyboard inputs, and is more frequent among adolescents with a history of STB [32]. More broadly, text-based linguistic markers identified via social media data or browser searches preceding acute mental health crises highlight the potential utility of these features to identify periods of acute suicide risk among youth [33–35].

Despite these advances, several critical gaps limit the translational utility of linguistic markers for predicting acute suicidal episodes in youth. First, most prior work has lexicon-based methods (e.g., LIWC or domain-specific dictionaries), which may fail to capture nuanced and context-dependent cues [36, 37]. Especially in the context of informal youth smartphone text that often includes slang, emojis, abbreviations, and short phrases [38], more advanced approaches, including topic models and transformer-based models may better detect nuanced linguistic features [39]. Recent work [40], including preliminary findings specifically among youth smartphone text [41] suggests that transformer-models may be more accurate compared to lexicon methods, and reveal clinical differences not identified by lexicons. Second, few studies have focused on identifying language patterns that precede acute suicidal crises (e.g., suicide attempts, emergency department visits, or psychiatric hospitalization) within short windows of time (e.g., hours to days). Understanding whether and how linguistic signals change during these proximal periods offers important implications for intervention development. Third, little is known about how algorithmically derived linguistic features align with expert clinical judgment [42, 43]. Although NLP methods offer scalability, clinical interpretation remains the gold standard in risk assessment and treatment, and direct comparisons could help clarify the practical utility and limitations of computational tools [34].

The present case studies seek to address these gaps by analyzing smartphone keyboard data from five adolescents during the 30 days leading up to a suicide-related hospitalization. Case studies are a common approach used to identify and characterize the validity of within-person data associations [44–48]. The current case studies of individuals with acute suicidal episodes leverage deep, within-person analyses (e.g., spanning thousands of text entries per individual) and clinician-generated annotations to identify the strengths and weaknesses of current NLP tools. First, we assessed the feasibility and quality of passively collected text data and characterized trends in sentiment and suicide-related language, including variation by time of day. We specifically compared these NLP signals between periods of relative health and more acute risk periods consisting of 10 days before hospitalization, based on previous work identifying changes in suicide risk factors within 1 to 2 weeks of attempts [7, 8, 15]. Second, we applied NLP topic models to examine individual-level trajectories of more nuanced linguistic themes throughout the 30 days. Last, we compared model-derived features to clinician-generated annotations to identify converging insights, missed signals, and directions for improving future models. Our overarching goal is to illuminate how passive text data and NLP can contribute to high-resolution understanding of suicide risk and inform future applications in larger, more generalizable youth samples.

## Materials and methods

### Study information

This study includes novel data analysis of cases from a completed research study with high-risk adolescents (N = 223) [5, 49], over-sampled for STB. Participants installed the Effortless Assessment Research System (EARS) app on their personal smartphone [50], which collected keyboard input data as well as other passive signals over a 6-month period. The keyboard input data provides signals of passive language and was the focus of the current study (see *Passive Language Data*). Assessments of sociodemographic information, comprehensive clinical history, psychiatric service use (e.g., hospitalization or emergency department visits for suicide-related concerns), and STB were administered at baseline, 1-month, 3-month and 6-month timepoints (see Text S1 for details). Study procedures were approved by the New York State Psychiatric Institute Institutional Review Board.

### Case selection

As the focus of the current study was on periods of acute risk, we included any participants who had language data within thirty days of hospitalization for STB (N = 5). Relative to general suicide behaviors, suicide-related hospitalizations were of interest as admittance dates could be confidently localized to an exact day using two sources–service use interviews and smartphone language data.

### Case details

Three female cases and two male cases (all identifying as cisgender) were selected. Information from self-reported demographics, SITBI [51] and service use questionnaires is shown in Table 1. We also include select clinical context (risk factors, study attempt method) from readings of deidentified smartphone language in the 30 days leading up to hospitalization. All cases had lifetime history of depressive and anxiety disorders, as well as histories of at least one lifetime suicide event prior to baseline (4 cases made previous attempts; 1 reported a plan and subsequent hospitalization). Endorsement of NSSI and methods of attempts varied.

**Table 1:** Case Characteristics and Language Data.

*Case Characteristics*			
*Case*	*Demographics*	*Clinical Profile*	*Hospitalization*
1	16, M, white, household income > 100k, NYC	GAD, behavioral conduct disorder, *history of MDD*, *history of substance use disorder*	Suicidal intent and risk of violence to others
2	17, F, multiracial, household income < 25k, PITT	MDD, GAD, multiple methods of NSSI, *history of MDD*	Suicide attempt - overdose
3	16, F, white, household income < 25k, PITT	MDD, other anxiety disorder^+^, repeated hospitalizations prior to study, *history of MDD*	Suicide attempt - poisoning*
4	18, M, white, household income > 100k, PITT	MDD, other anxiety disorder^+^. Access to firearms. Extensive NSSI (cutting). Online help-seeking. *History of MDD*.	Suicide attempt - hanging
5	14, F, white, household income > 100k, PITT	MDD, GAD, specific phobia, OCD, *history of MDD*	Suicide attempt (method unclear)

*Smartphone Text Entries*					
*Case*	*Total Entries*	*Entries/Day 30 d*	*Entries/Day 10 d*	*Days with Entries*	*Entry MNW*
1	11,942	110.87	86.82	134	3.62
2	24,381	145.19	168.09	162	6.54
3	40,077	213.13	143.82	140	4.40
4	25,669	207.71	271.73	110	4.39
5	3005	83.45	235.18	32	4.41

Clinical profiles report current diagnoses and past diagnoses (‘*History of’*).

*M* male, *F* female, *SES* socioeconomic status, *GAD* generalized anxiety disorder, *MDD* major depressive disorder, *OCD* obsessive-compulsive disorder, *NSSI* non-suicidal self-injury, *NYC* New York City, *PITT* Pittsburgh.

Total Entries: recorded entries across the entire study period, after preprocessing. Entries/day 30 d: mean entries per day for 30 days before hospitalization. Entries/day 10 d: mean entries per day for 10 days before hospitalization (acute periods). Entry MNW: mean number of words per entry. *Poisoning: self-consumption of non-medication (bleach, laundry detergent).^+^other anxiety disorder: anxiety disorder other than GAD.

### Passive language data

Smartphone keyboard inputs were passively acquired by the EARS app, which was developed and maintained by Ksana Health [23]. Each keyboard input (all characters typed in any app), was timestamped and labeled with the app used, then inputs were transformed into text entries using an algorithm identifying logical divisions (e.g., pauses, changes in app). These text entries typically consisted of messages, searches, posts, or notes and were only recorded as typed by the participants. Thus, this did not include incoming messages from other individuals with whom participants may have been interacting. All text entries then underwent a multi-layered de-identification process to protect participant privacy. Custom in-house algorithms by Ksana Health team removed sensitive personal information such as passwords, credit card numbers, and other structured sensitive data formats. Following this initial pass, we used Microsoft Presidio, an open-source, automated de-identification tool, to detect and redact personally identifiable information (PII), including names, geographic locations, phone numbers, email addresses, and other common identifiers [52]. Further processing is reported in Text S2.

### EARS data modalities

We focused on language data to narrow the scope of this study. Consistent with prior findings within the broader MAPS cohort [53], experience sampling data were frequently missing, with data only available on an average of 32.7% (SD = 33.8%) of the days preceding and including hospitalization (fig. S1). Among passive modalities, keyboard input data had the highest availability (M = 96.4%, SD = 5.0%), whereas sleep data meeting quality criteria (M = 9.1%, SD = 20.3% available, fig. S2) and GPS data (M = 78.2%, SD = 23.7% available, fig. S3) were missing more frequently.

### Case methodology: overview

We used a mixed-methods framework including qualitative thematic coding of text prior to hospitalization, as well as automatic NLP metrics. Primarily, we used visual graphical analysis of the extracted measures. We focused on a ten-day window before hospitalization as an ‘acute risk period,’ as this represents a period during which clinical intervention and bridging to services may be provided. For automated NLP, we illustrated signals for this period as well as the period twenty days prior, which we called the ‘baseline period’ (i.e., prior to acute suicide risk). Sensitivity analyses to the acute risk window were conducted, consisting of selecting different length windows from 1 to 30 days before hospitalization and comparing NLP signals to the study means. Case Study 5 was hospitalized ten days after beginning the study, and thus, we were only able to examine language from the acute period.

### Clinical risk extraction

Two licensed clinicians (SS, KD) with extensive experience in adolescent mental illness and STB extracted events/ factors that may confer increased suicide risk. The purpose of this analysis was to highlight day-level events of interest by reviewing deidentified text entries, and context across text entries, in the ten-day window before hospitalization. These sets of individualized risk events were compared to automated NLP. Events and their days of occurrence were extracted according to a set of 17 categories which was iteratively developed during coding: for example, interpersonal conflict, substance use, and academic stress (Text S3).

### NLP measures

#### Suicide-related language

To identify suicide-related text entries, a youth suicide lexicon was deployed [32]. The lexicon combined key word detection, slang adaptations, multi-word flagging, and emoji detection. This lexicon, adapted from prior published work [31, 37, 54], was recently shown to identify suicide-related language with high recall (sensitivity) and to differentiate adolescents with STB history. Thus, the proportion of suicide-related language per day was examined as a risk predictor for hospitalization. This lexicon does not distinguish types of suicide-related language (e.g., active intent versus jokes or hyperbole). A lexicon was chosen based on the lack of validated transformer models for youth suicide language.

#### Negative sentiment

To identify text entries expressing negative sentiment, a transformer-based model (*tweetnlp*) was deployed [40]. Transformer models are based on using the sophisticated multi-scale predictive model using linguistic units to predict language to follow, making them sensitive to the context of a given phrase, sentence, or set of sentences. Even when constrained to individual text entries, this context sensitivity confers better sentiment identification (Text S4**)**. Thus, the proportion of negative messages per day was examined as a risk predictor for hospitalization. On an exploratory basis, we classified negative messages as negative self-reference if they included first-person pronouns.

#### Topic modeling

Topics were identified through similarity-based embeddings using the BERTopic package [55]. We specifically applied supervised topics in BERTopic, which consisted of 10 possible topics (e.g., treatment, family, sleep), with seed words used to identify messages that were similar to those topics (Text S5). The frequency of topics per day was assessed.

### Visual graphical analysis

A graphical analysis was used to facilitate human interpretation of NLP metrics like suicide language and sentiment during risk periods. The main visualization involves plotting automated NLP metrics during the baseline period (−30 to −10 days) and acute period (−10 days to hospitalization). Metrics were centered relative to the mean of the NLP metric over the ***entire study*** for that participant to identify changes in language from one’s typical patterns. The sensitivity of the metric, and thus suitability as a risk indicator, is reflected in whether there are visually detectable increases or decreases during the acute period. Likewise, specificity is reflected in whether there are increases or decreases also observed during the baseline period. Both determinations are suggestive in the absence of statistical models.

A secondary visual analysis was conducted to compare NLP measures and clinician risk event timelines. The goal was to assess whether clinical events (e.g. substance use or STB) by clinician annotators were also captured by NLP (e.g., the topic of substance use, or the STB lexicon). Timelines were placed on the same axes as the NLP measures and detailed inspection was conducted. To optimize accessibility, we have summarized the results of this coding in the main text, while supplying example comparison plots (e.g., STB lexicon versus timelines).

## Results

### Data quality and missingness

Language data were present on a high proportion of days preceding hospitalization; however, the quantity of entries as well as lengths of entries varied by participant (Table 1). On average over the baseline and acute periods, each participant reported at least 75 text entries per day, providing sufficient data for NLP.

### Negative sentiment and suicide-related language

**Fig. 1:**
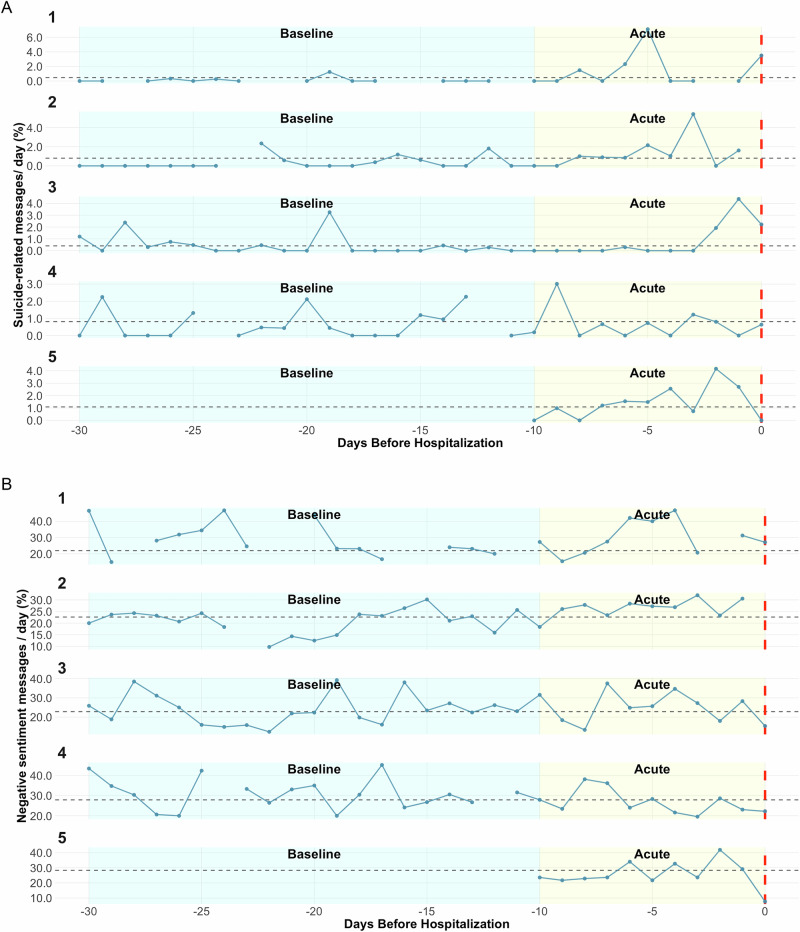
Negative sentiment and suicide-related language before hospitalization in all cases. Blank days reflect missing data. **A** suicide related language is shown; (**B**), the proportion of messages expressing negative sentiment per day is shown. The black horizontal dashed line represents the participant-specific mean across the entire study period. The red vertical line represents the day of hospitalization (Day 0). Per methods, we have divided the period up into an acute phase (10 days prior) and a baseline phase (30 – 10 days prior). the y-axis differs for each panel to make relative differnces clear. Case 5 was hospitalized only 10 days after starting the study, therefore no baseline period exists for this participant.

**Fig. 2:**
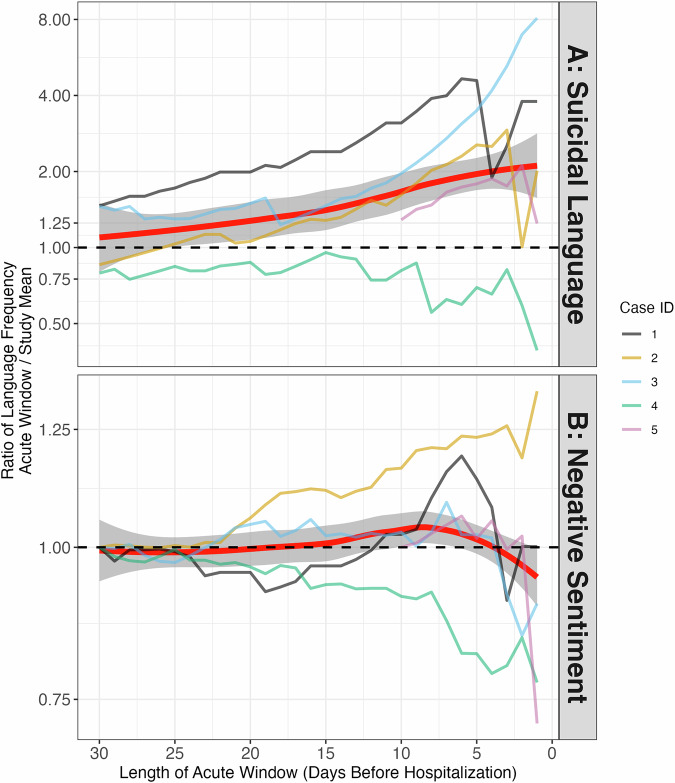
Elevations in suicide language and sentiment are most pronounced in windows of 0–10 days pre hospitalization. The y-axis shows the within-person ratio of the frequency of suicidal language (**A**) and negative sentiment (**B**) during acute windows of varied length relative to the mean frequency during the entire study (e.g., a ratio of 2 indicates twice the frequency of language during a given acute period relative to the entire study). Acute window lengths, shown on the x-axis, encompass the N days prior to hospitalization (including the day of hospitalization). **A** Suicide language is shown. **B** Negative sentiment is shown. Red thick line and shaded confidence region represents loess fit at the group level, and thin lines represent individual participants. The black dashed line reflects equivalence between acute window and study mean (ratio of 1). Case 5 was hospitalized only 10 days after starting the study, as such the window does not exceed 10. Ratios are significantly larger for suicide language, partially reflecting small base rates.

Across 4 of the 5 individuals (excepting Case 4), both negative sentiment and suicide-related language increased during the acute period (10 days prior to hospitalization, Fig. 1) relative to their person-specific mean frequencies. Increases in proportion of suicide-related language were notable. For example, Case 3 showed an 11-fold increase on the day prior to hospitalization. Increases were more modest for negative sentiment (Fig. 2), although absolute changes in entries were larger as the base rate of negative sentiment was substantially higher (> 25x). Analyses of sensitivity to different acute period lengths for negative sentiment and suicide-related language revealed that most pronounced escalations in suicide language occurred within 5 days of hospitalization, and that more diffuse increases in negative sentiment were found (within 10 days) (Fig. 2). When examining negative *self-referential* messages (fig. S4), increases were also found for Cases 1–3.

### Individual-specific signals: time periods

We examined language use in four time periods (morning, afternoon, evening, midnight). Some participants showed frequent smartphone entries after midnight, while no post-midnight entries were collected for others (Supplement fig. S5). When examining negative sentiment and suicide language changes in the four time periods, we observed varying patterns across individuals (Supplement fig. S6**)**. Finally, we noted that suicidal language increases were most pronounced on weekends in the acute period, and that negative sentiment increases were most pronounced on weekdays (Supplement fig. S7).

### Individual-specific signals: topics

**Fig. 3:**
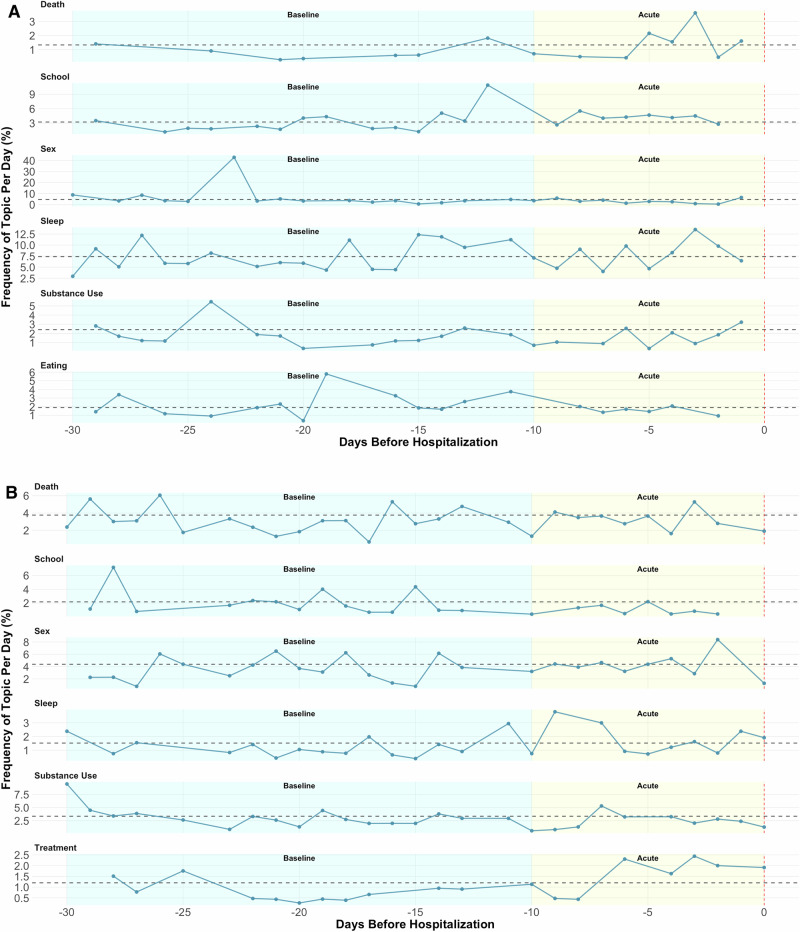
Topic frequency trajectories for two cases. **A** Topic trajectories for Case 2; (**B**) Topic trajectories for Case 4. Black dotted line represents mean frequency over the entire study. The same 5 topics (sex, sleep, school, death, substance use) were selected for both, and treatment and eating, on bottom, were selected based on individual-specific prevalence.

Frequencies of supervised topics varied over time and across participants. For example, Case 4 showed an increase in treatment-related entries (e.g., medication, psychiatry) during the acute period, while Case 2 showed little to no discussion throughout the periods (Fig. 3). On the other hand, Case 2 discussed eating frequently, albeit in decreasing frequency throughout the acute period, while language about school and death increased in the acute period (Fig. 3B**)**. No topics appeared robustly sensitive (increasing) in the acute period across participants, highlighting their individual-specific nature.

### Clinician coding of events

**Fig. 4:**
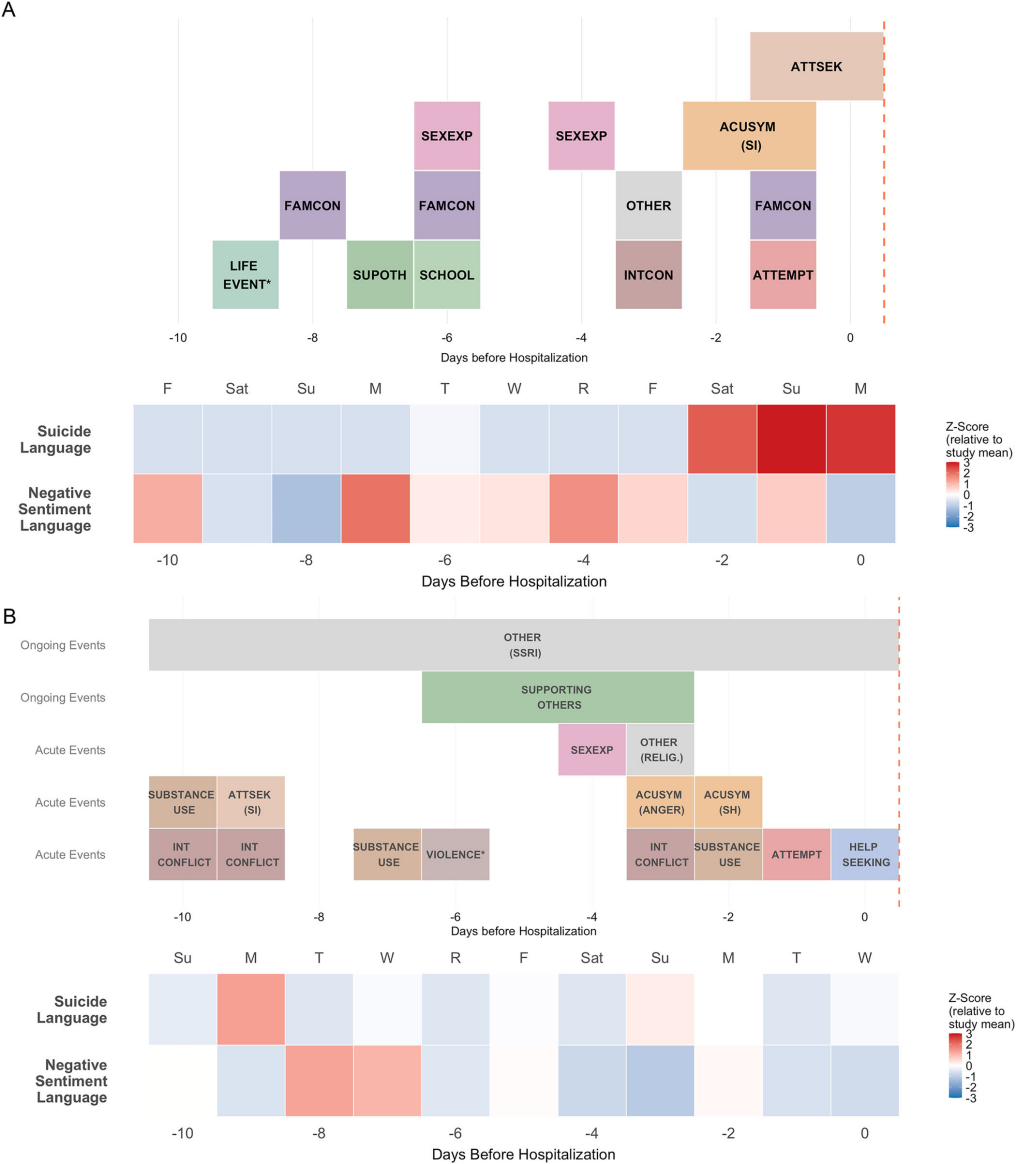
Timelines and NLP metrics for Cases 3 and 4. Colors represent different categories of risk events, and red-blue heatmap represents frequency of suicide language and negative sentiment language as detected by NLP. These plots were used to assess correspondence of NLP metrics with timeline, via observing overlaps between increases in frequency of language and events. Asterisks denote events that are explained further. **A** Timeline for Case 3. ACUSYM (SI): refers to suicidal ideation; SEXEXP: sexual experience, FAMCON: family conflict, INTCON: interpersonal conflict, SUPOTH: reflects supporting others, ATTSEK: attention-seeking, SCHOOL: academic stressor, OTHER: deleting social media accounts. LIFE EVENT*: refers to future move. **B** Timeline for Case 4. OTHER (RELIG.) reflects religious language; OTHER (SSRI) reflects changes in SSRI medication. VIOLENCE* refers to an episode of online threatening language. SI: suicidal ideation; SH: self-harm. Timelines were created in R4.5.0 with categories indicated by color coding, and separate rows used to indicate coincident events [66].

Clinical coding of smartphone language data revealed a variety of events during the acute period (Fig. 4; fig. S8-21). Some participants’ language showed frequent substance use or substance seeking (Cases 1, 4), whereas others described online victimization (Case 2) or discussed school stressors (Cases 2, 3). Expressions of interpersonal and family conflicts were common, as well as sexual behavior online or in-person. Clinicians noted language that clearly indicated suicidal behavior; Cases 2–5 all made attempts on the day before hospitalization, and Case 1 expressed active suicidal ideation and may have had a violent family conflict the day prior. In addition, clinicians also noted some uses of suicidal language reflecting a desire to gain attention from others by shocking friends or acquaintances (Cases 1, 3, 4), other communication reflecting explicit distress (all cases), and still others directly seeking help or support (all cases). Communication could reflect strategies for coping with STB or the use of STB language to meet other psychological needs. Finally, many participants also took active roles in supporting vulnerable friends—in some cases, the risky or concerning behavior of friends seemed to have negatively influenced the participant’s own mental health (Case 4, 5).

#### Correspondence with NLP measures of sentiment and suicidal language

Among the current cases, clinical coding of salient events during the acute period did not correspond with increases in the frequency of negative sentiment, even on days where clinicians indicated ‘acute symptoms’ (Fig. 4, S8-S21). On the other hand, peaks in suicidal language often did correspond in time with salient events (Fig. 4, S8-S21). Sometimes those peaks occurred on days of attempts (Cases 1,3,5) or suicidal ideation preceding attempts (All Cases). In some cases, suicidal language also may have reflected help-seeking after the attempt (Cases 1, 3). With Case 4, clinicians noted STB in a friend of the participant, but because there was no explicit STB language, it was not detected by the lexicon.

#### Correspondence with NLP measures of topics

Certain topics tended to correspond more with clinically coded events (fig. S8–21). Increased language about substance use, and discussion of treatment generally co-occurred with events of the same nature. Other topics more variably overlapped with events depending on whether the topics were highlighted for negative sentiment or frequency (e.g., school overlapping with academic stressors, and death overlapping with ideation). Topics that showed little to no correspondence with potential events included family, friends and sleep. These topics are more abstract, have fewer prototypical words, and tended to show less segregated embeddings (fig. S22). Due to the diffuseness of these topics, clinician-coded interpersonal and family conflicts were largely missed by NLP.

## Discussion

Temporally sensitive methods to identify increased STB risk remain limited, particularly outside of healthcare contacts. In the current case study of five adolescents hospitalized for suicide risk, NLP methods showed sensitivity to risk periods, with all individuals experiencing increases in suicidal language, and 4 of 5 individuals showing sustained increases above the mean in negative sentiment prior to hospitalization. Suicidal language increases often corresponded to clinician judgments of suicidal ideation, attempts, and help-seeking. Topic models often captured increased frequency of language about substance use and treatment. Despite these promising findings, significant gaps between NLP metrics and the nuanced, time-sensitive, and person-specific insights from clinical judgements emerged. Thus, a central question for future research involves how to integrate context and personal insight into NLP methods.

To our knowledge, our study was the first to report the sensitivity of NLP methods applied to passively sensed smartphone language data to uncover signals of distress in adolescent suicidal crises. NLP methods have previously identified signals of distress within-individuals [29–31, 47], but have largely relied on rule-based approaches that may not fully capture constructs of interest (e.g., misclassifying sentiment). In the current study we used an extensively validated lexicon for adolescent suicidal language [32] as well as transformer embedding methods (but not LLMs). The methods incorporated in these studies may prove integral to improve sensitivity to risk signals. Both methods are readily deployed, identity-protective, and cost-effective, and could be incorporated in future statistical modeling applications.

Although sensitivity matters, specificity to suicide risk is equally important given limited clinical resources and the potential for stigma and/or medical mistrust following unnecessary interventions [56]. In the current study, specificity was assessed by examining signals during baseline periods vs acute risk periods. The observational nature of the study means that differences herein, whether detected by NLP or clinicians, may not be causal for the suicide-related hospitalization. Though the specificity of increased suicide-related language to the acute period was higher, baseline periods sometimes included increases in both suicide-related language and negative sentiment **(**Fig. 1**)**, which likely reflects distress but may not presage imminent suicide risk. Further, there were pronounced elevations in suicidal language in the several days prior to hospitalization (i.e., 1–5 days prior), while elevations in negative sentiment were more temporally diffuse (Fig. 2), suggesting that specific NLP features may correspond to different time windows of risk. For this reason, we also examined supervised topic modeling to detect individual-specific events before hospitalization.

Topic models uncovered increased frequency of certain topics prior to hospitalization (e.g., treatment, sleep, and school), but the correspondence to clinical reports was often lacking and depended on how salient topics were identified (e.g., frequency vs sentiment of topics). Clinicians often identified periods of interpersonal conflict that were not detected by topic models, which could be potential triggers for STB [57, 58]. For example, Case 4 had a friend who engaged in extremely risky behavior, and this may have contributed to their own escalation of NSSI and STB. Relatedly, feelings of peer rejection and exclusions appeared to meaningful contribute to increased risk for Case 5. Clinicians also identified differences in communication for help relative to communication for attention-seeking (e.g., suicide gestures) which, in some cases, may differentiate the severity of distress. This distinction gave them insight into how individuals coped with distress. For example, Case 2 showed a clear shift from positive appraisals of a sexual experience to negative and self-shaming perspectives. Topic models often lacked context to detect these kinds of changes.

It should be noted, however, that evidence for and against associations between NLP metrics and STB are limited by the case approach. For example, it may be that negative language about sleep is a consistent risk factor for suicide across people, but not so strongly associated that it is clear in a sample of five individuals. Future statistical modelling in larger samples could more comprehensively establish which NLP metrics are associated with risk.

### Limitations: improving individual-specific models of risk

Incorporating context (e.g., diagnostic or clinical history, prior language history) could provide better individual-specific signals of risk. The NLP methods deployed only incorporate context implicitly, in the sense that metrics are displayed relative to a within-person average. The implications of this process may be seen in the topic and sentiment analyses: if a participant sends ‘I went to school’ and ‘I hated it’ in separate consecutive messages, our current methods will not detect the common context. Ideally, the two would be combined to infer ‘negative sentiment about school’. Future approaches should combine signals from the sentiment and content of language (e.g., by expanding model input to multiple messages) to provide more nuanced representations of risk factors.

Another fundamental issue concerns the window of risk. Here we examined ten days as our acute risk period because of its relevance to taking preventative clinical actions. This also facilitated visual analysis and is concordant with other passive sensing findings highlighting risk factors specific to the 1–2 weeks prior to suicide events [15]. It is possible, however, that events occurring outside this period influenced eventual hospitalization [33], or that changes immediately preceding attempts were most influential [59, 60]. Indeed, when examining the timing of individual elevations in suicide and negative sentiment language (Fig. 2), we found significant individual heterogeneity (although all peaks excluding case 4 occurred within 10 days of hospitalization). Future well-powered studies incorporating statistical modeling could examine NLP signals across and within individuals, incorporating various time windows of predictors (e.g., t-1 week, t-2 weeks) and testing time-lagged associations between negative sentiment and suicide language.

Finally, it is possible that multimodal passive sensing may provide greater specificity. Other work by our group has identified links between both GPS-derived time spent at home and passively detected sleep with the likelihood of next-week suicide events [15, 17]. It is likely that individual signals from language, GPS, and sleep represent constrained risk factors, which when combined may lead to escalation [12, 61]. In the current study, language data was more available than these other signals, although Case 5 had substantially less language data than the others which may have limited detection of changes relevant to hospitalization. Future studies should assess the feasibility of multimodal prediction of suicidal thoughts and behaviors.

### Applications

NLP detection of short-term suicide risk factors could facilitate digital treatment approaches and just-in-time interventions, which may be especially helpful for individuals without access to other clinical services [62, 63]. These methods applied to passively sensed language data could help to resolve discrepancies between youth behaviors and self-reports to adapt customized treatments for specific individuals. Timely information about salient topics and sentiment could inform clinical care. Future work will also need to address critical ethical issues around digital surveillance [64, 65], and information about the content of smartphone communication would need to be masked suitably to protect privacy (perhaps by using language models to represent topics). Results from our group’s studies with vulnerable youth suggest that conscientious and safeguarded digital data collection is highly acceptable [53], but understandably, for some families and circumstances this may not be the favored approach. Future work could examine the effects of smartphone interventions with and without the use of automated signals to inform clinical utility.

### Summary

The present study examined the promise and challenges of using automated NLP methods with smartphone data to detect short-term suicide risk. Current NLP methods work well for identifying clear warning signs in single text entries, such as suicide-related language or negative sentiment. However, they are ineffective at recognizing individual risk factors—like conflict with friends or family—that are informed by context across many entries. Progress in this area will likely depend on new AI and other methods that can take this context into account and integrate language with other forms of passive data.

## Supplementary information

Supplemental Material

## Data Availability

Data from the Mobile Assessment for the Prediction of Suicide Study are shared via the NIMH Data Archive (NDA ID #3502) at https://nda.nih.gov/edit_collection.html?id=3052. Given the sensitive nature of text data and the possibility that they may not be fully deidentified even after removing names, phone numbers, and other identifiers, raw text cannot be shared publicly to ensure compliance with participants’ consent. Code is shared at https://github.com/itreves36/NLP_Hospitalization.
