# Supplementary material for: In their own words: case studies of adolescent smartphone language preceding suicide-related hospitalizations
Source: NPP Digit Psychiatry Neurosci. 2026 Mar 2;4:5. doi: 10.1038/s44277-026-00057-0 (PMC12953750; doi:10.1038/s44277-026-00057-0)
Supplement: Supplementary file 1 — Supplemental Material [file 44277_2026_57_MOESM1_ESM.docx]

**Supplemental Materials** for **In Their Own Words: Case Studies of Adolescent Smartphone Language Preceding Suicide-related Hospitalizations**

Isaac N. Treves^1+,^, Paul A. Bloom^1+^, Samantha Salem^1^, Katherine Durham^1^, Valerio Zaccaria^1,2^, Jamaal Spence^1^, Peter S. Dayan^3^, Lauren S. Chernick^3^, Ashley Blanchard^3^, Jaclyn S. Kirshenbaum^1^, Esha Trivedi^1,8^, David A. Brent^4^, Nicholas B. Allen^5^, Jamie Zelazny^6^, Karla Joyce^7^, Giovanna Porta^7^, David Pagliaccio^1,8^, and Randy P. Auerbach^1,8^

^+^co-first authorship

**Affiliations:**

^1^Department of Psychiatry, Columbia University

^2^Department of Human Neuroscience, Sapienza University of Rome, Italy

^3^Department of Emergency Medicine, Columbia University

^4^Department of Psychiatry, University of Pittsburgh Medical Center

^5^Department of Psychology, University of Oregon

^6^School of Nursing, University of Pittsburgh

^7^Western Psychiatric Hospital, University of Pittsburgh Medical Center

^8^Division of Child and Adolescent Psychiatry,New York State Psychiatric Institute

Corresponding author: Isaac N. Treves, [isaac.treves@nyspi.columbia.edu](mailto:isaac.treves@nyspi.columbia.edu)

Table of Contents

[Supplemental Methods 3](#_Toc218172598)

[Supplement Text 1: Assessments and Case Selection 3](#_Toc218172599)

[Supplement Text 2: Additional Information on Keyboard Input Preprocessing 3](#_Toc218172600)

[Supplement Text 3: Instructions 3](#_Toc218172601)

[Supplement Text 4: Transformer Models 4](#_Toc218172602)

[Supplement Text 5: Supervised Topics 5](#_Toc218172603)

[Supplemental Figures: Other Mobile Sensing 6](#_Toc218172604)

[Figure S1: EMA data around dates of hospitalization 6](#_Toc218172605)

[Figure S2: Usable EARS sleep data 6](#_Toc218172606)

[Figure S3: Usable homestay data around dates of hospitalization. 7](#_Toc218172607)

[Supplemental Figures: Negative Self-referential Language 7](#_Toc218172608)

[Figure S4: Negative self-referential messages/day. 8](#_Toc218172609)

[Supplemental Figures: Time of Day 9](#_Toc218172610)

[Figure S5: Communication by Time of Day 9](#_Toc218172611)

[Figure S6: 10](#_Toc218172612)

[Supplemental Figures: Weekdays and Weekends 11](#_Toc218172613)

[Figure S7: 11](#_Toc218172614)

[Supplemental Figures: Risk Events 12](#_Toc218172615)

[Figure S8: Timeline, Sentiment, Suicide Language Correspondence Plot for Case 1. 13](#_Toc218172616)

[Figure S9: Timeline and Topic Frequency Plot for Case 1. 14](#_Toc218172617)

[Figure S10: Timeline and Topic Sentiment Plot, Case 1. 15](#_Toc218172619)

[Figure S11: Timeline, Topic, Sentiment Plot for Case 2. 16](#_Toc218172620)

[Figure S12: Timeline and Topic Frequency Correspondence Plot, Case 2. 17](#_Toc218172621)

[Figure S13 Timeline and Topic Sentiment Correspondence Plot, Case 2. 18](#_Toc218172622)

[Figure S14: Timeline, Sentiment, and Suicide Language Correspondence Plot, Case 3. 19](#_Toc218172623)

[Figure S15: Timeline and Topic Frequency Correspondence Plot, Case 3. 20](#_Toc218172624)

[Figure S16: Timeline and Topic Sentiment Correspondence Plot, Case 3. 21](#_Toc218172626)

[Figure S17: Timeline, Sentiment and Suicidal Language Correspondence Plot, Case 4. 22](#_Toc218172627)

[Figure S17: Timeline and Topic Frequency Correspondence Plot, Case 4. 23](#_Toc218172629)

[Figure S18: Timeline and Topic Sentiment Correspondence Plot, Case 4. 24](#_Toc218172630)

[Figure S19: Timeline, Sentiment and Suicidal Language Correspondence Plot, Case 5. 25](#_Toc218172632)

[Figure S20: Timeline and Topic Frequency Correspondence Plot, Case 5. 26](#_Toc218172634)

[Figure S21: Timeline and Topic Sentiment Correspondence Plot, Case 5. 27](#_Toc218172635)

[Figure S22: Embeddings for Case 1. 28](#_Toc218172636)

[References 29](#_Toc218172637)

# Supplemental Methods

## Supplement Text 1: Assessments and Case Selection

At baseline, participants provided sociodemographic information and a comprehensive clinical battery including the Mini International Neuropsychiatric Interview for Children and Adolescents, Version 7.02 (MINI-KID; Sheehan et al., 1998), a structured interview assessing Diagnostic and Statistical Manual of Mental Disorders (Fifth Edition) psychiatric disorders in adolescents. Additionally, at baseline participants were administered the Self-Injurious Thoughts and Behaviors Interview (SITBI; Nock et al., 2007) to assess the lifetime, past-year, past-month, and past-week presence, frequency, and severity of self-injurious thoughts and behaviors. At each follow-up, adolescents reported on STB since the past assessment. If indicated by clinical interviews or experience sampling, the Columbia Suicide Severity Rating Scale (C-SSRS; Posner et al., 2011) was administered to assess imminent STB risk. If imminent risk was detected, staff bridged participants to clinical services. Participants also completed a structured interview at each assessment regarding psychiatric service utilization (e.g., psychiatric hospitalization or emergency department visits for suicide-related concerns).

As described previously, all participants who had suicide-related hospitalizations were included in the case study. Relative to general suicide behaviors, suicide-related hospitalizations were of interest as admittance dates could be confidently localized to an exact day using two sources—service use interviews and smartphone language data (i.e. whether they reported a hospitalization in text entries from their phone). The one exception is 5, where an initial attempt is confirmed by the SITBI and smartphone language, but the following hospitalization was only reported in smartphone language. Overall, we are confident in separating periods before and after hospitalization with day-level accuracy.

## Supplement Text 2: Additional Information on Keyboard Input Preprocessing

Names of public and historical figures who died by suicide were retained in the text during the de-identification step. Text was further preprocessed: blank entries, system-generated text, URLs, entries with only a single character (other than emojis), and duplicate entries within the same app with timestamps separated by less than 1s were discarded. Spell-correction was performed using a HIPAA-compliant instance of GPT3.5 for all entries. Spell-correction was used for automated NLP, while a version of the text without spell-correction was supplied to the human coders, so as to preserve the initial communication as much as possible.

## Supplement Text 3: Instructions

We extracted events relevant to the suicide-related hospitalization of the individual, as informed by the clinical expertise of SS and KD, as well as literature on known factors for STB^4,5^. Events may be acute (e.g., one-time substance use) or extended over multiple days (e.g. online bullying). Events were reported in a neutral frame (e.g., ‘substance use’, not ‘stimulating substance use’). The following themes based on author consensus were used to categorize the events, as well as deidentify them.

We categorized the events according to the same themes across all participants:

- Substance use (SUBUSE)
- Interpersonal conflict (INTCON)
- Family conflict (FAMCON)
- Suicide Attempt (ATTEMPT)
- Self-report Acute symptoms (e.g. extreme anxiety, suicidal ideation, or self-harm) (ACUSYM)
- Sex-related events or sexual experiences (SEXEXP)
- Online victimization (CYBVIC)
- Academic stressors (SCHOOL)
- Violence (VIOLEN)
- Supporting other, e.g. giving advice about depression or suicidal thoughts to a friend (SUPOTH)
- Help-seeking (HLPSEK)
- Attention-seeking: language about distress or emotionality that may be intended to shock or communicate distress without direct requests for help (ATTSEK)
  - Suicidal gestures
  - Sexually provocative
  - Violent language
- Transition/ Life Event (LIFE EVENT)
- Other (OTHER)

Color Schemes for timeline:

**Substance use:** #D4B5A0 (muted beige-brown) **Interpersonal conflict:** #C5A3A3 (dusty rose) **Family conflict:** #B8A8C8 (soft lavender) **Attempt:** "#E8A6A6” (red) **Self-report acute symptoms:** #F0C49A (pale peach) **Sex-related events:** #E6B3CC (muted pink) **Online victimization:** #9DB4C0 (soft blue-gray) **Academic stressors:** #B8D4B8 (pale sage green) **Violence:** #C8B5B5 (warm gray-pink) **Supporting other:** #A8C8A8 (gentle mint) **Help-seeking:** #B8C8E6 (soft periwinkle) **Attention-seeking:** #E6C8B8 (muted peach-tan) **Sleep/awake patterns:** #C8C8D4 (light gray-blue) **Other:** #D8D8D8 (very light gray), **Transition/ Life Event**: #B3D4C7 (dark mint)

## Supplement Text 4: Transformer Models

Context-sensitive NLP is particularly important in the case of adolescent passive language, which is highly heterogeneous and involves slang. We have found that this is particularly important for sentiment models. For example, “you are killing it!” is positive but less context-aware models may identify this as negative given the word ‘killing’. In an in-preparation manuscript using a test-set of 1500 hand-coded entries, a transformer model from tweetnlp^6^ performed significantly better than rule-based approaches like VADER (F1_tweetnlp_ = 0.74, F1_VADER_ =0.58). Thus, in the current study, we used a fine-tuned *tweetnlp* model based on a hold-out set of 840 hand-labeled messages from the entire cohort (the neural network weights across the transformer were optimized to produce the lowest loss on 840 messages balanced across sentiment classes).

Despite the added sensitivity of the transformer models to context, we chose to focus on text entries individually. This was mainly conducted so that we could construct continuous measures of negative sentiment (e.g., daily frequency of negative sentiment), as opposed to determining whether a given period of text was negative (e.g. was the last hour negative). Other reasons include a history of previous validation on individual text entries or tweets,^7,8^ and qualitative observations that many text entries are disjoint and could result in contradictions when combining them. As described, even in the case of individual messages, context matters (e.g., ‘my head is killing me’ vs. ‘you killed it on the test’) and can explain why *tweetnlp* out performs VADER).

##

## Supplement Text 5: Supervised Topics

seed_topic_list = [

sleep: ["sleep", "bed", "tired", "wake up", "can't sleep", "nap"],

eating: ["food", "hungry", "appetite", "eating", "meal"],

death: ["dying", "death", "dead", "kill", "pass away"],

school: ["teacher", "class", "school", "test", "grade"],

family: ["parents", "brother", "sister", "cousin", "family"],

friends: ["friend", "best friend" "girlfriend", "boyfriend", "partner"],

substance use: ["weed", "alcohol", "drugs", "cigarette", "smoke"],

sex: ["sex", "porn", "dick", "pussy", "horny"],

treatment: ["medication", "treatment", "therapy", "meds", "surgery"],

social media: ["instagram", "facebook", "discord", "twitter", "tiktok"]

]

Topics were selected based on a combination of preliminary unsupervised analyses which revealed common over-arching types of language use, which authors IT and JS then decided to constrain to the most common types and most common words. The orange words represent seed words that were used to identify language by embedding similarity, which was then categorized belonging to topic (white).

We chose to leave out traditional unsupervised topic analyses as they would be harder to implement in an automatized NLP pipeline (i.e., one needs the full data, a justifiable threshold for number of topics, and a way to label them and de-identify them).

# Supplemental Figures: Other Mobile Sensing


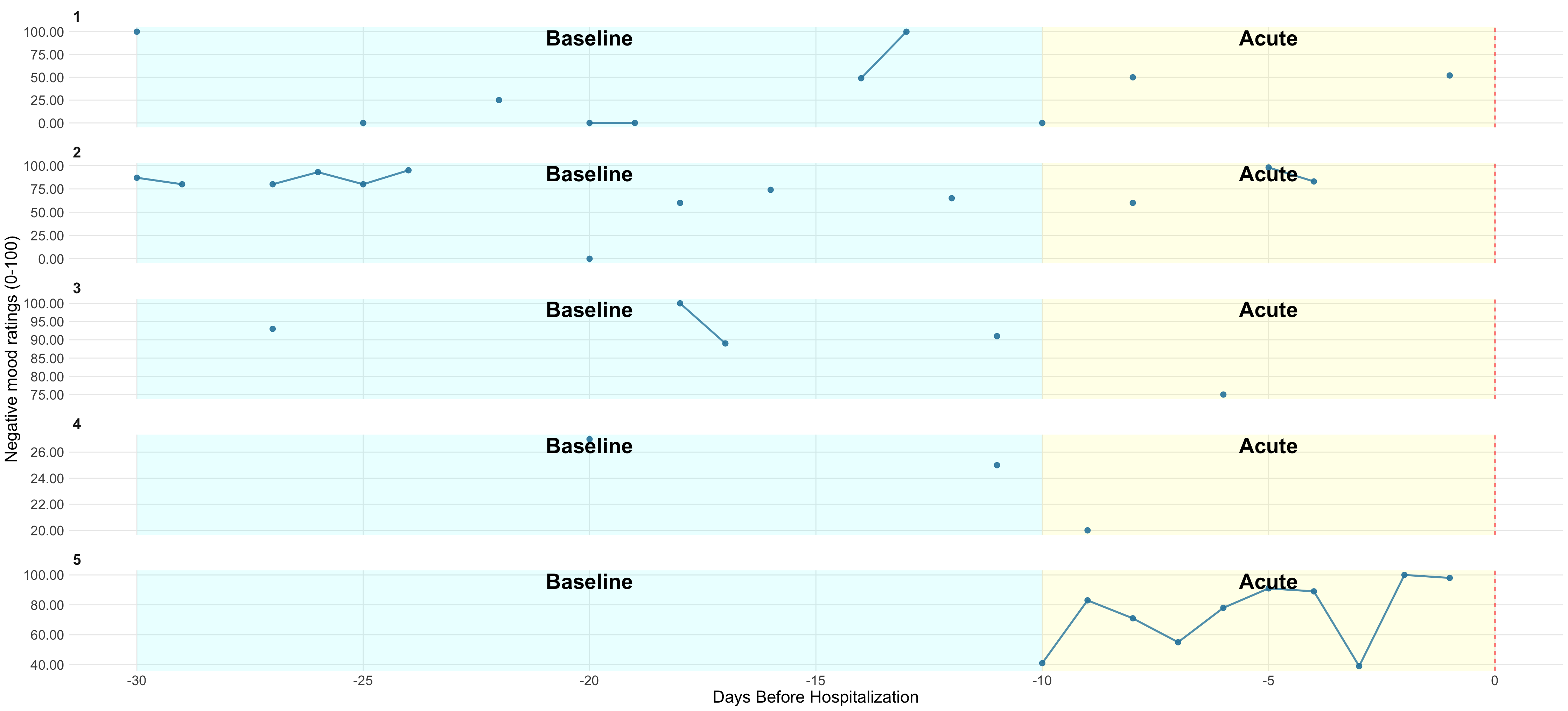


Figure S1: ESM data around dates of hospitalization, where higher ratings means more negative mood. The black line represents the mean across the entire study. The red line represents the day of hospitalization. Per methods, we have divided the period up into an acute phase (10 days prior) and a baseline phase (30 – 10 days prior). Case 5 started the study 10 days before hospitalization and as such is missing baseline data.

**
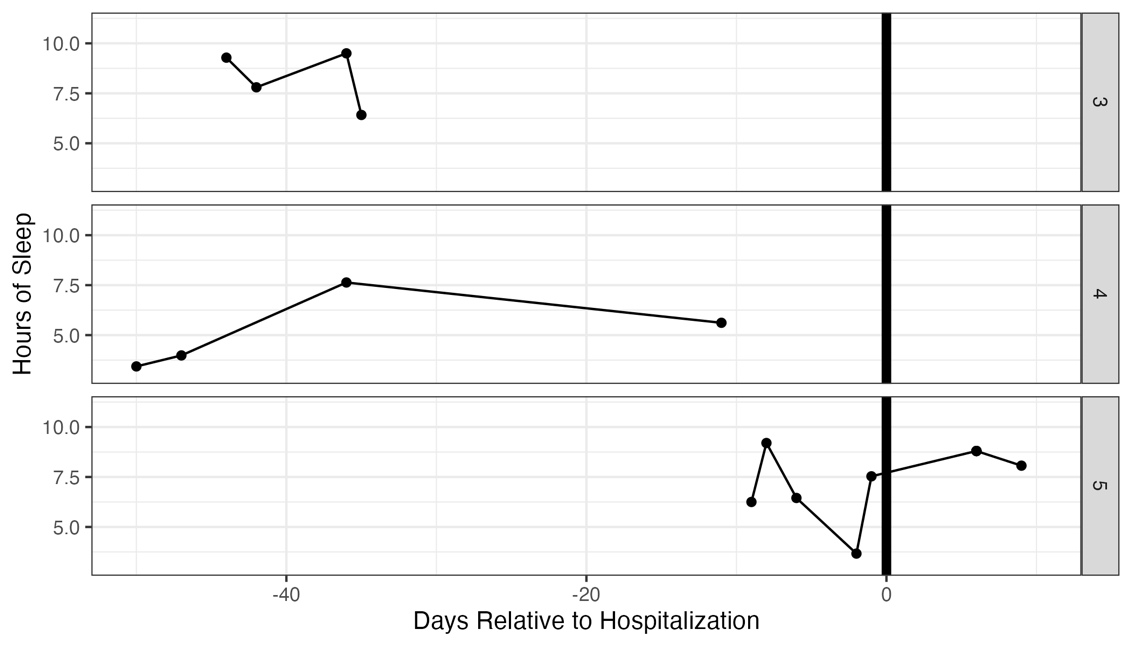
**

Figure S2: Usable EARS sleep data around dates of hospitalization (black line).

**
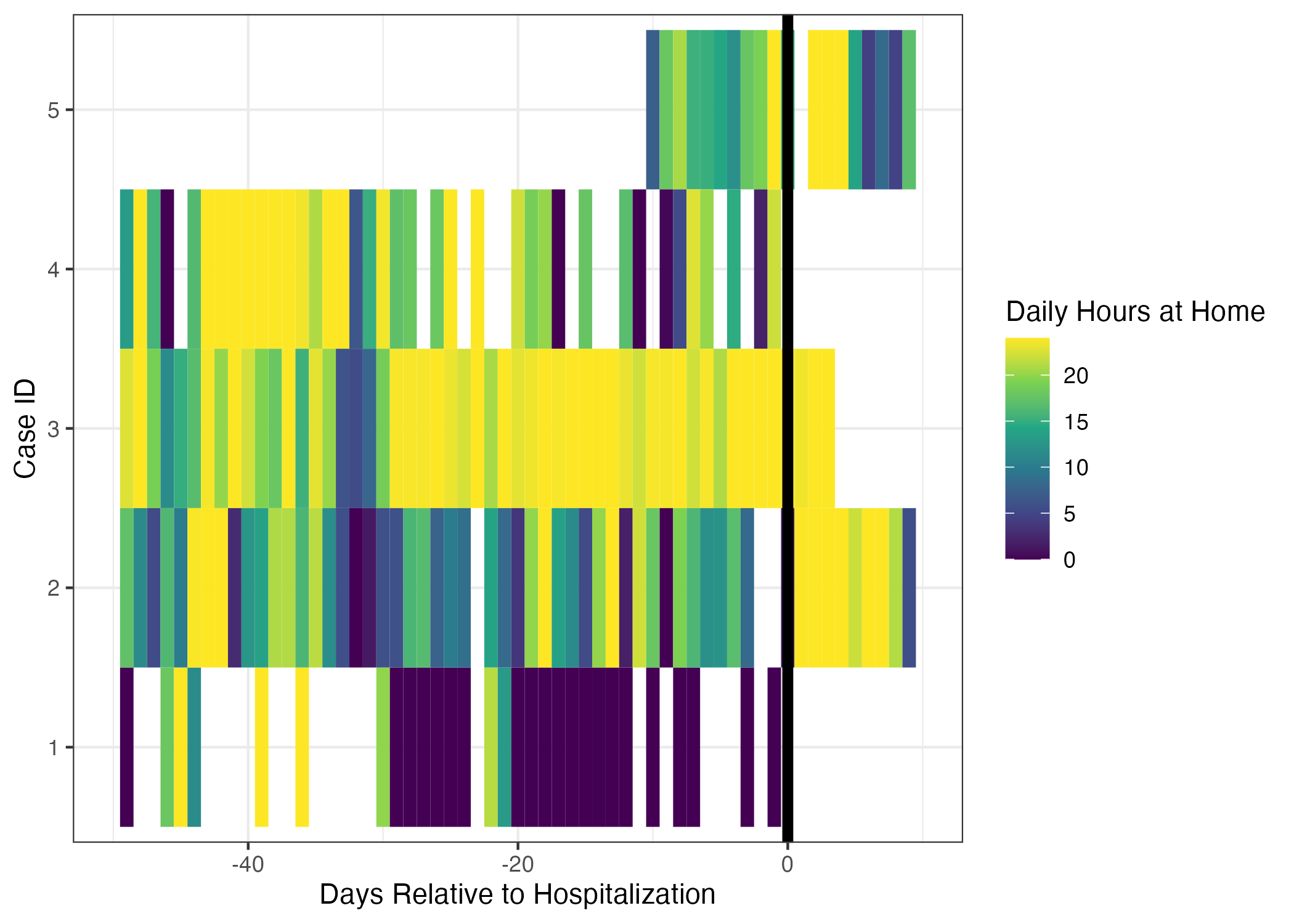
**

## Figure S3: Usable homestay data around dates of hospitalization.

# Supplemental Figures: Negative Self-referential Language


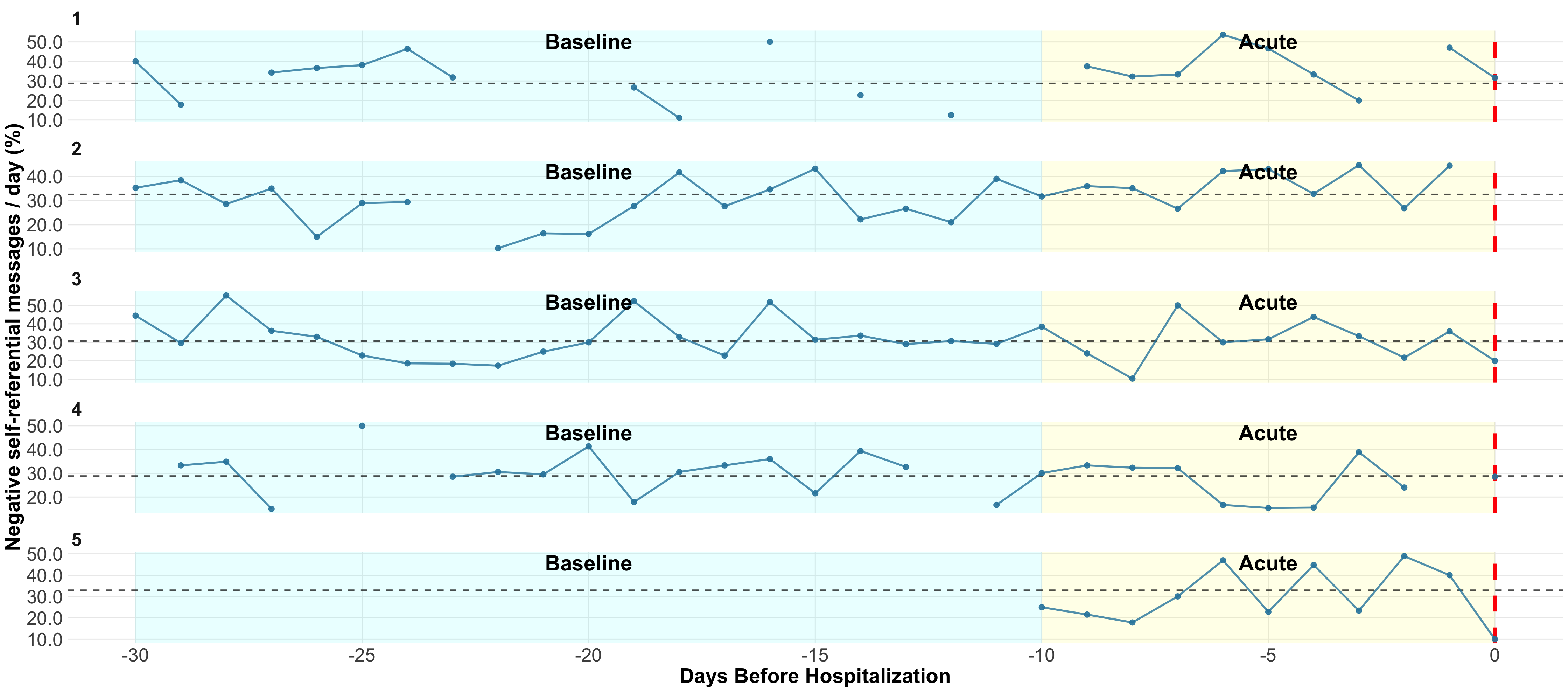


Figure S4: Negative self-referential messages/day. Blank days reflect missing data. The black horizontal dashed line represents the participant-specific mean across the entire study period. The red vertical line represents the day of hospitalization (Day 0). Per methods, we have divided the period up into an acute phase (10 days prior) and a baseline phase (30 – 10 days prior). Note: the y-axis differs for each panel to make relative differnces clear. Case 5 was hospitalized only 10 days after starting the study, therefore no baseline period exists for this participant.

#
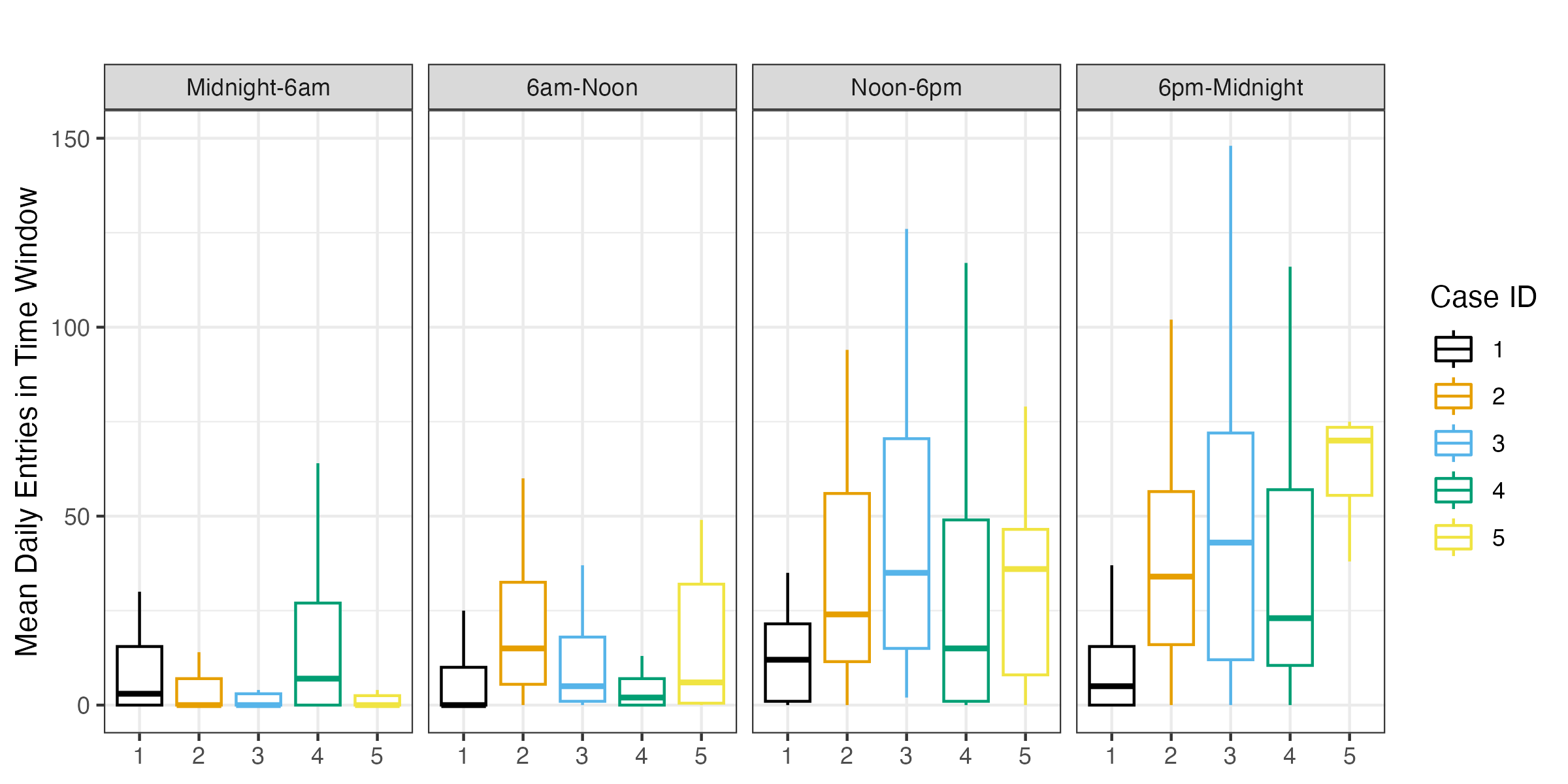
Supplemental Figures: Time of Day


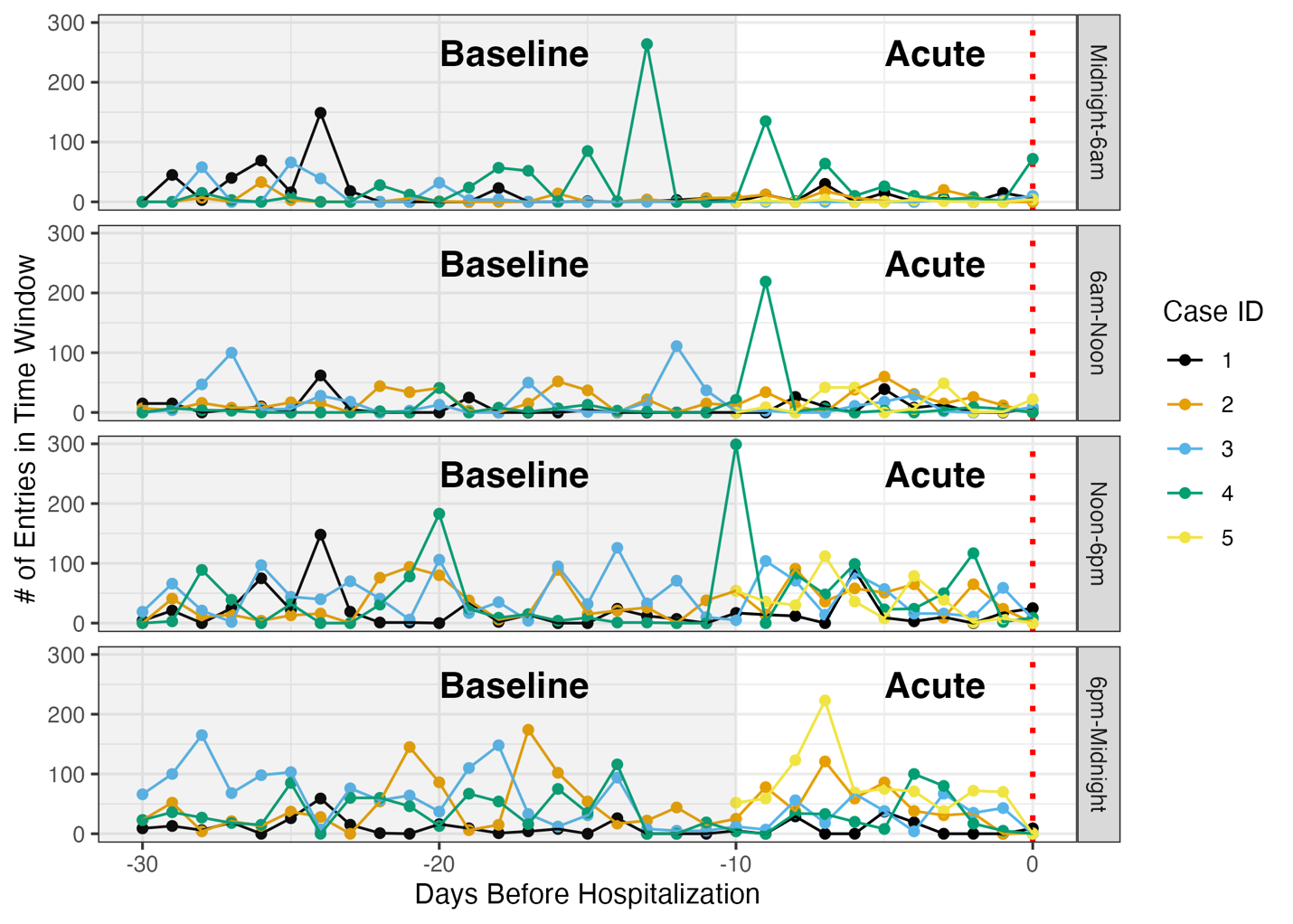
Figure S5: Communication by Time of Day (30 days up until hospitalization)


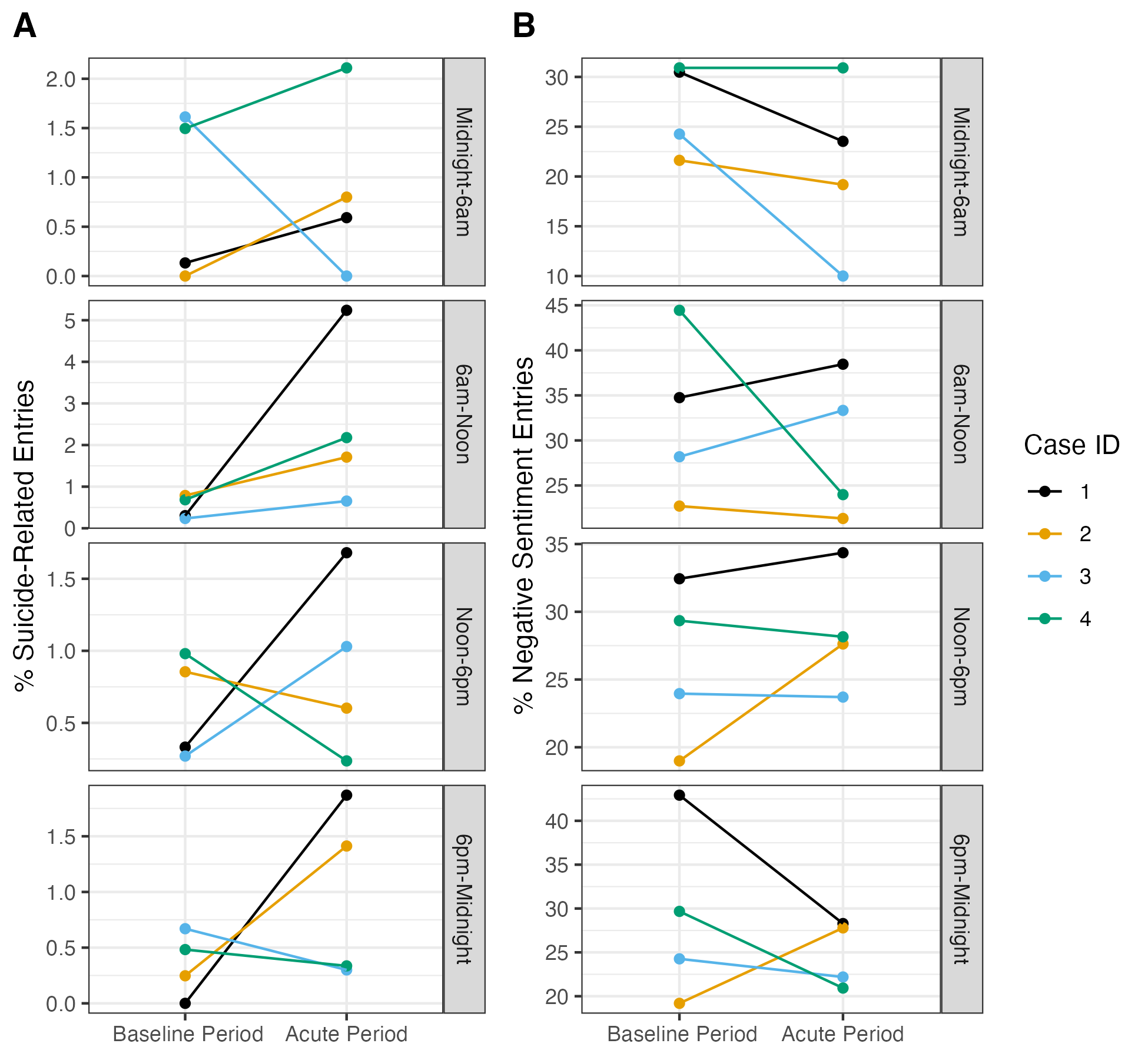
Figure S6: Negative sentiment and suicide language frequency changes from the baseline to acute period before hospitalization by time of day. Each row shows a time window (from top to bottom: after midnight = Midnight-6am, morning = 6am-Noon, afternoon = Noon-6pm, night = 6pm-Midnight). Y-axis values show the percentage of entries with negative sentiment (**Panel A**, left**)** and suicide-related language **(Panel B,** right) within each time window for the baseline period and acute period (x-axis). Each line represents one participant. Case 5 was hospitalized only 10 days after starting the study, therefore no baseline period exists for this participant and they were not shown in this figure.

# Supplemental Figures: Weekdays and Weekends


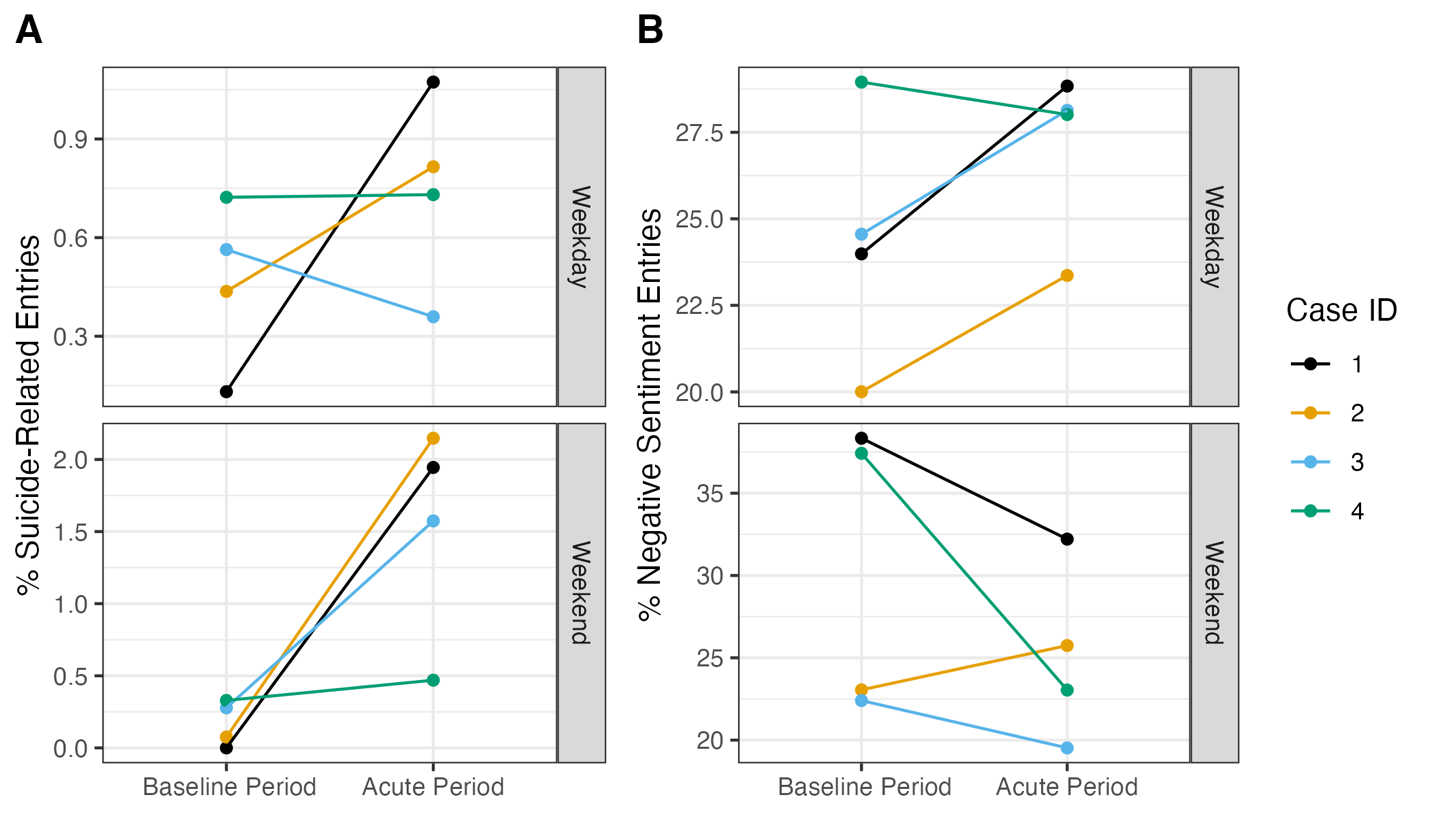


Figure S7: Average percentages of suicide-related entries (Panel A) and negative sentiment entries (Panel B) for each participant on weekdays (Monday through Friday) versus weekends (Saturday, Sunday). Case 5 started the study 10 days before hospitalization and as such is missing baseline data and not included in this figure. It may be observed that suicidal language increases were most pronounced on weekends in the acute period, and that negative sentiment increases were most pronounced on weekdays.

# Supplemental Figures: Risk Events


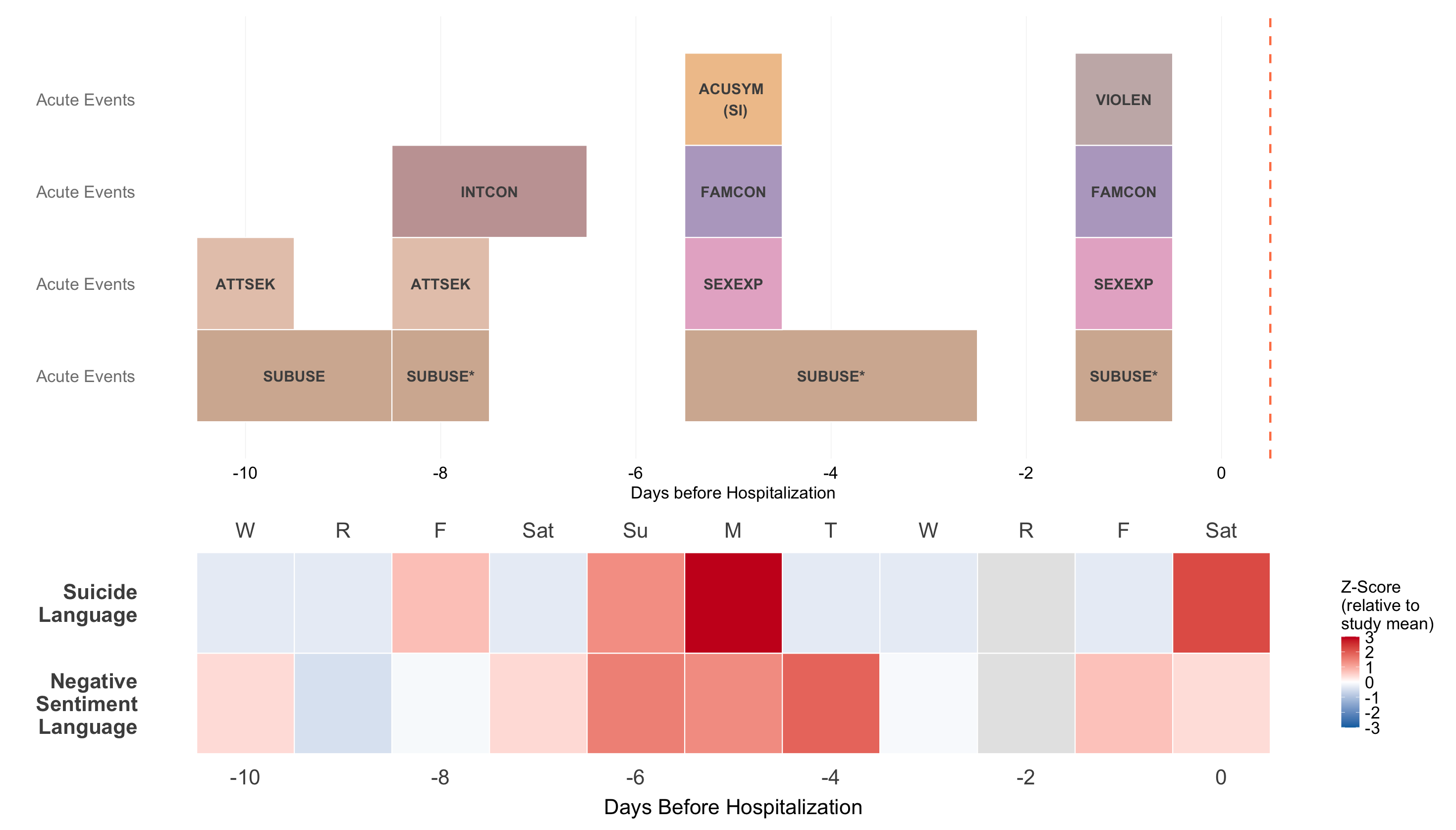


Figure S8: Timeline, Sentiment, Suicide Language Correspondence Plot for Case 1. SUBUSE*: refers to substance seeking.


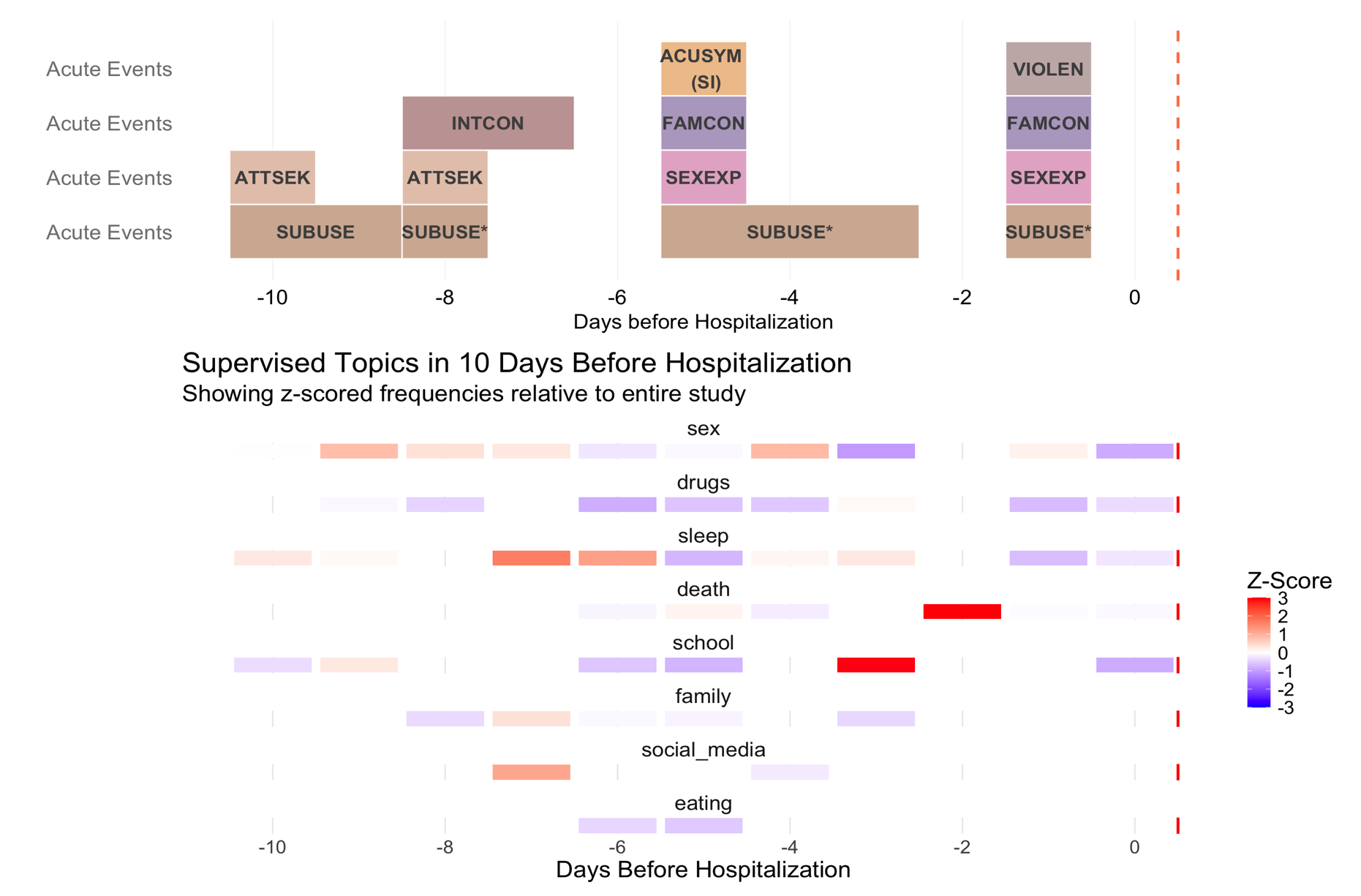


Figure S9: Timeline and Topic Frequency Plot for Case 1. SUBUSE*: refers to substance seeking.

##
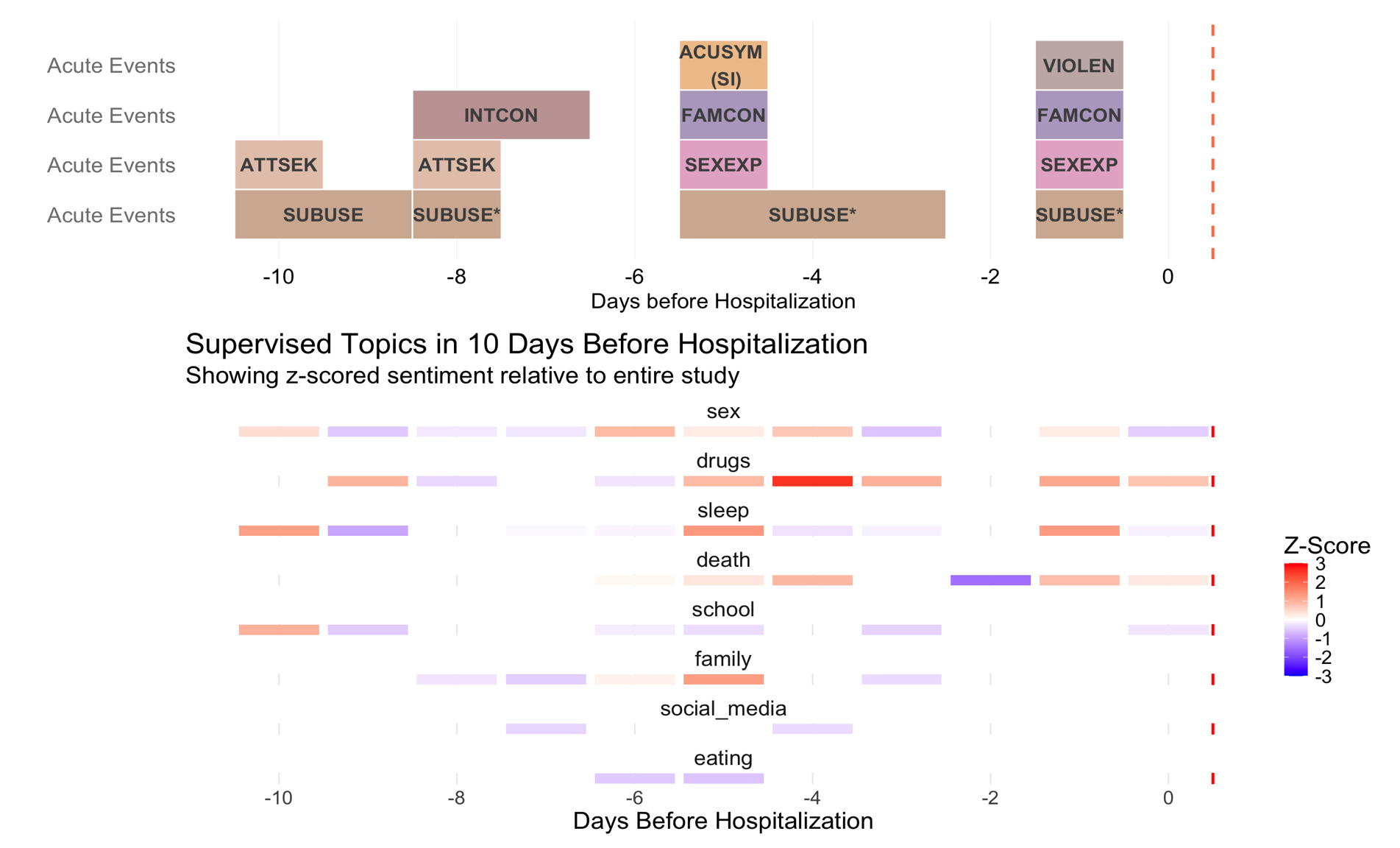
Figure S10: Timeline and Topic Sentiment Plot, Case 1.


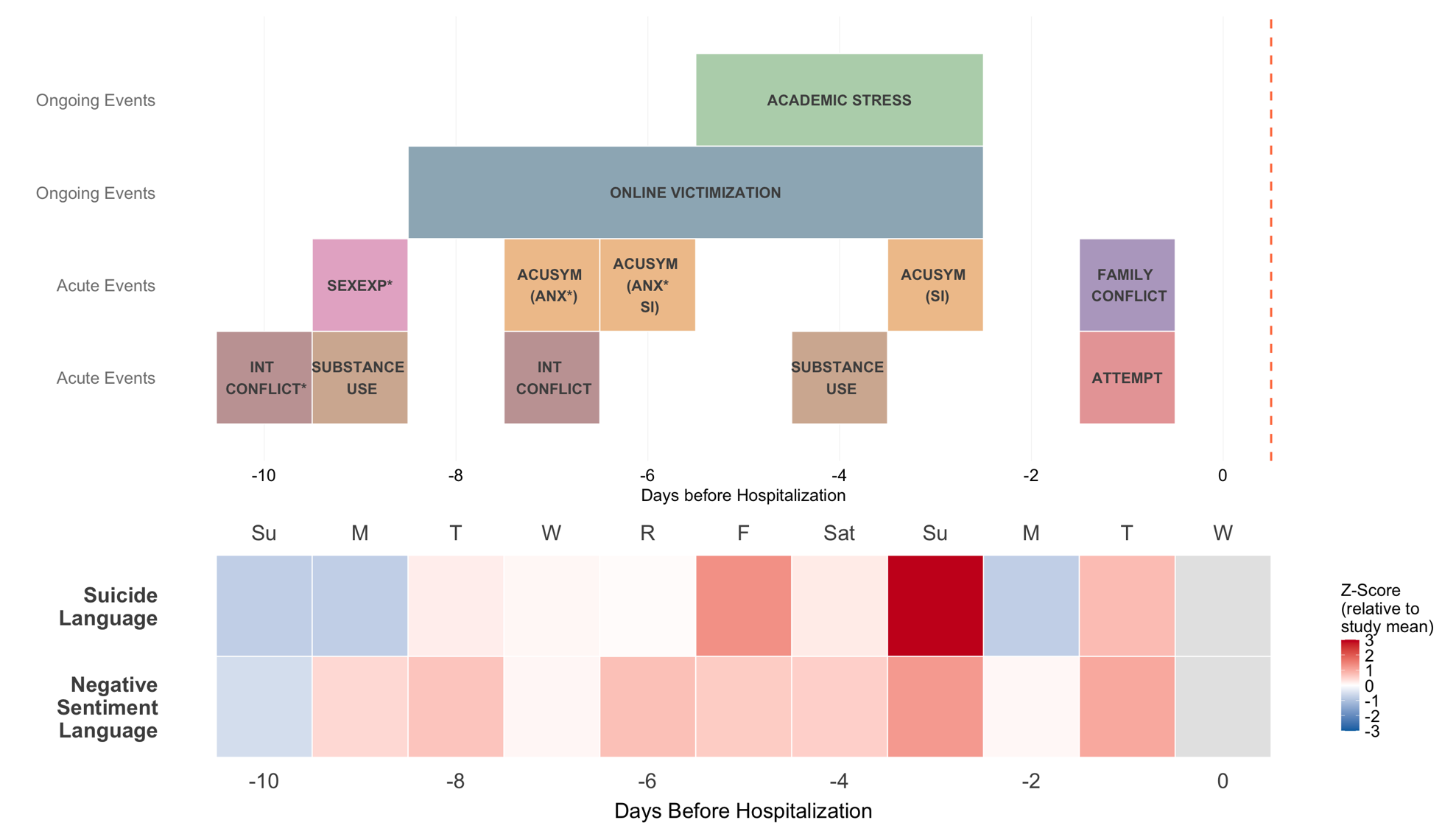


Figure S11: Timeline, Topic, Sentiment Plot for Case 2. ACUSYM (ANX*): refers to self-reported panic attacks; INTCON*: refers to romantic rejection; SEXEXP*: refers to a sexually stigmatizing experience.


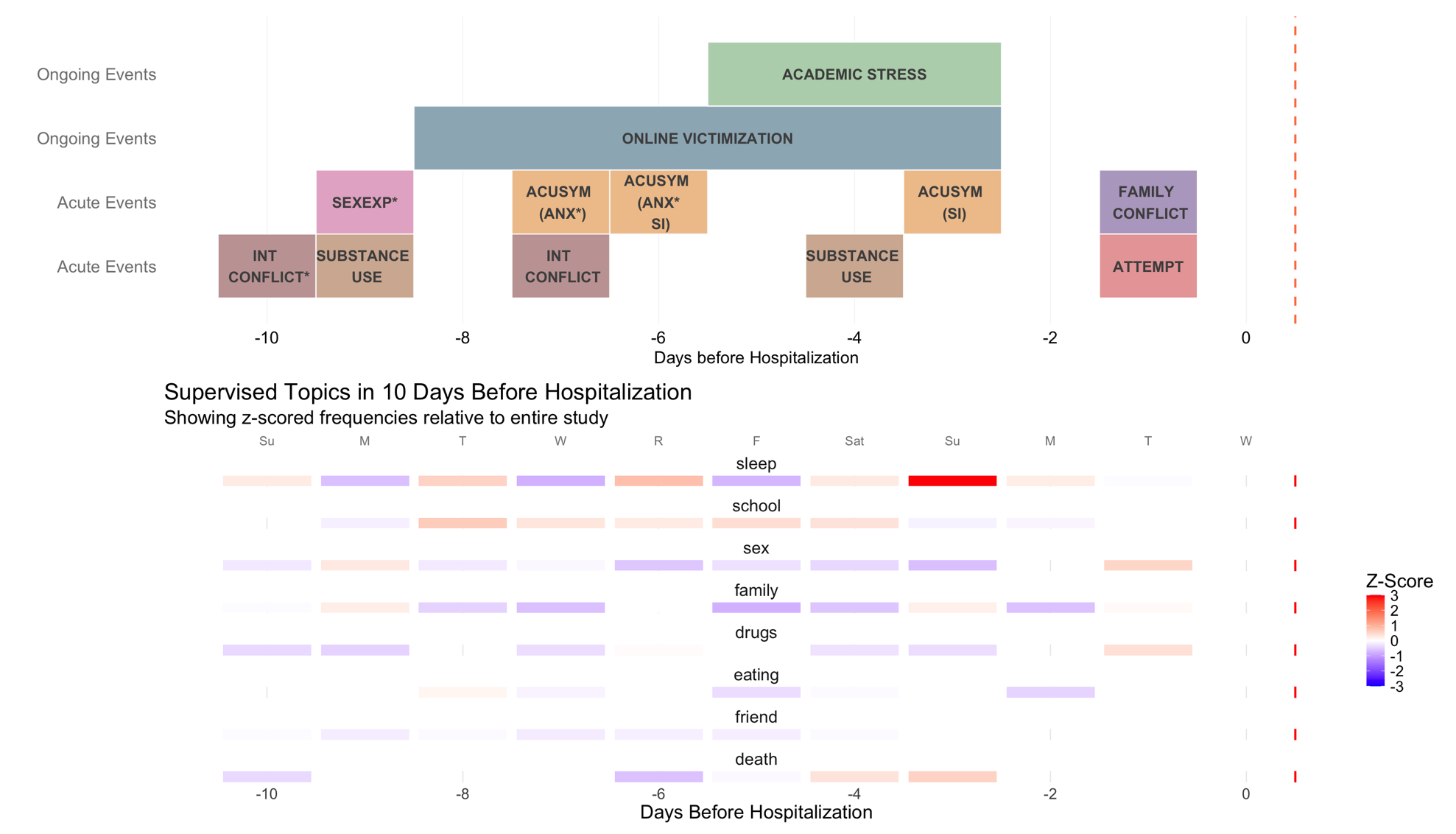


Figure S12: Timeline and Topic Frequency Correspondence Plot, Case 2. ACUSYM (ANX*): refers to self-reported panic attacks; INTCON*: refers to romantic rejection; SEXEXP*: refers to a sexually stigmatizing experience


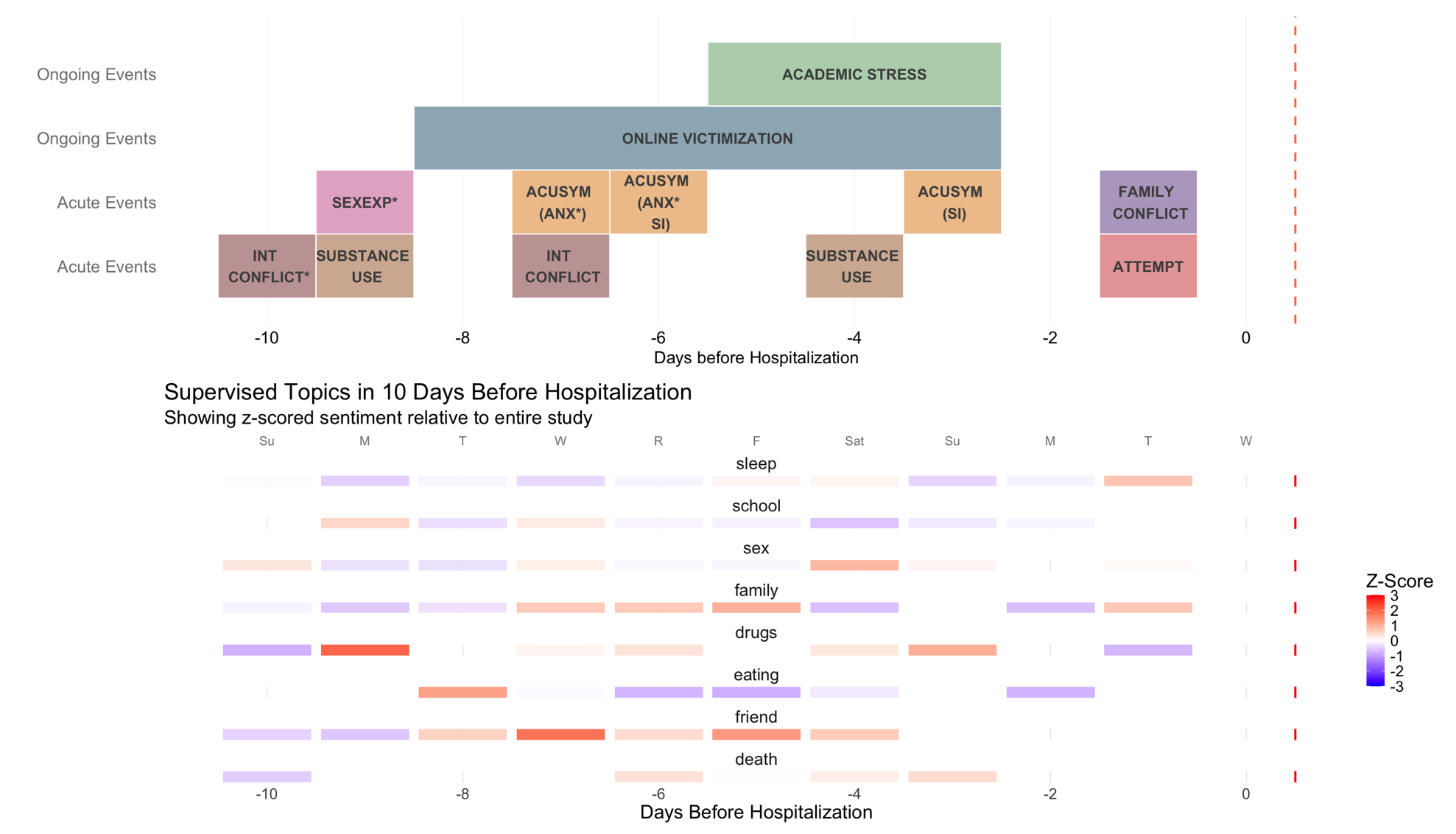


Figure S13 Timeline and Topic Sentiment Correspondence Plot, Case 2. ACUSYM (ANX*): refers to self-reported panic attacks; INTCON*: refers to romantic rejection; SEXEXP*: refers to a sexually stigmatizing experience


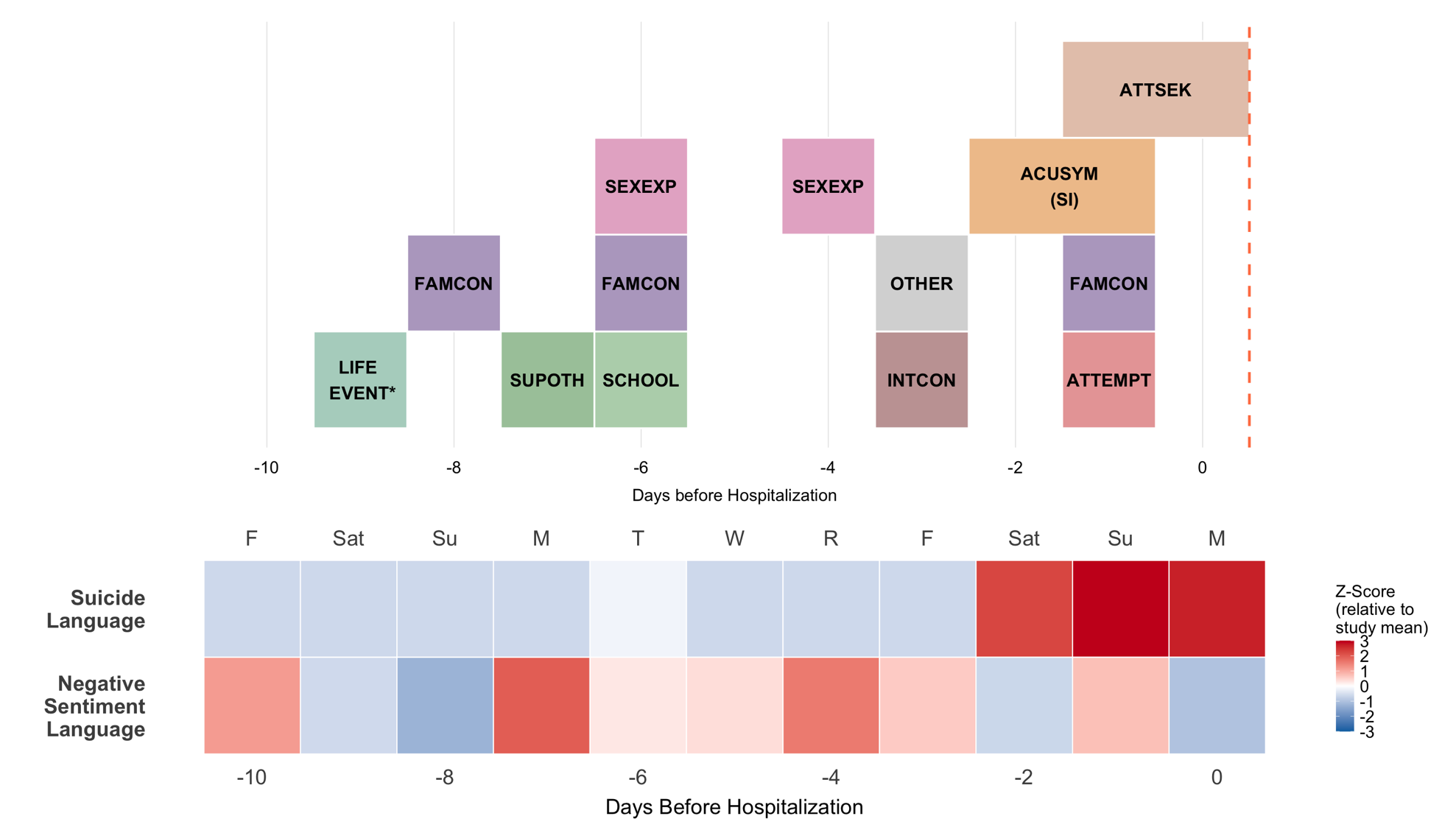
Figure S14: Timeline, Sentiment, and Suicide Language Correspondence Plot, Case 3. LIFE EVENT*: reflects discussion of a future move.


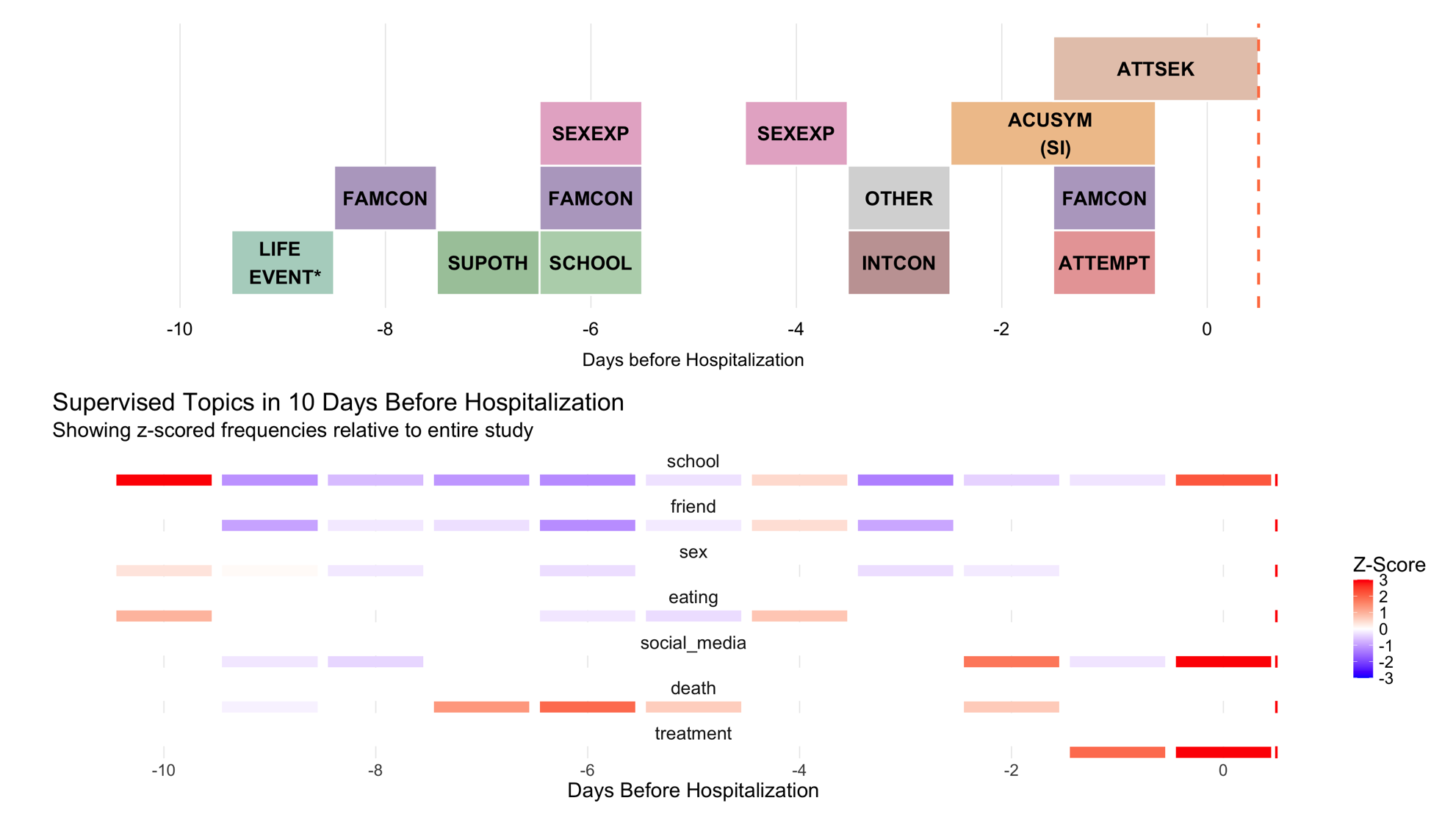


## Figure S15: Timeline and Topic Frequency Correspondence Plot, Case 3.

##
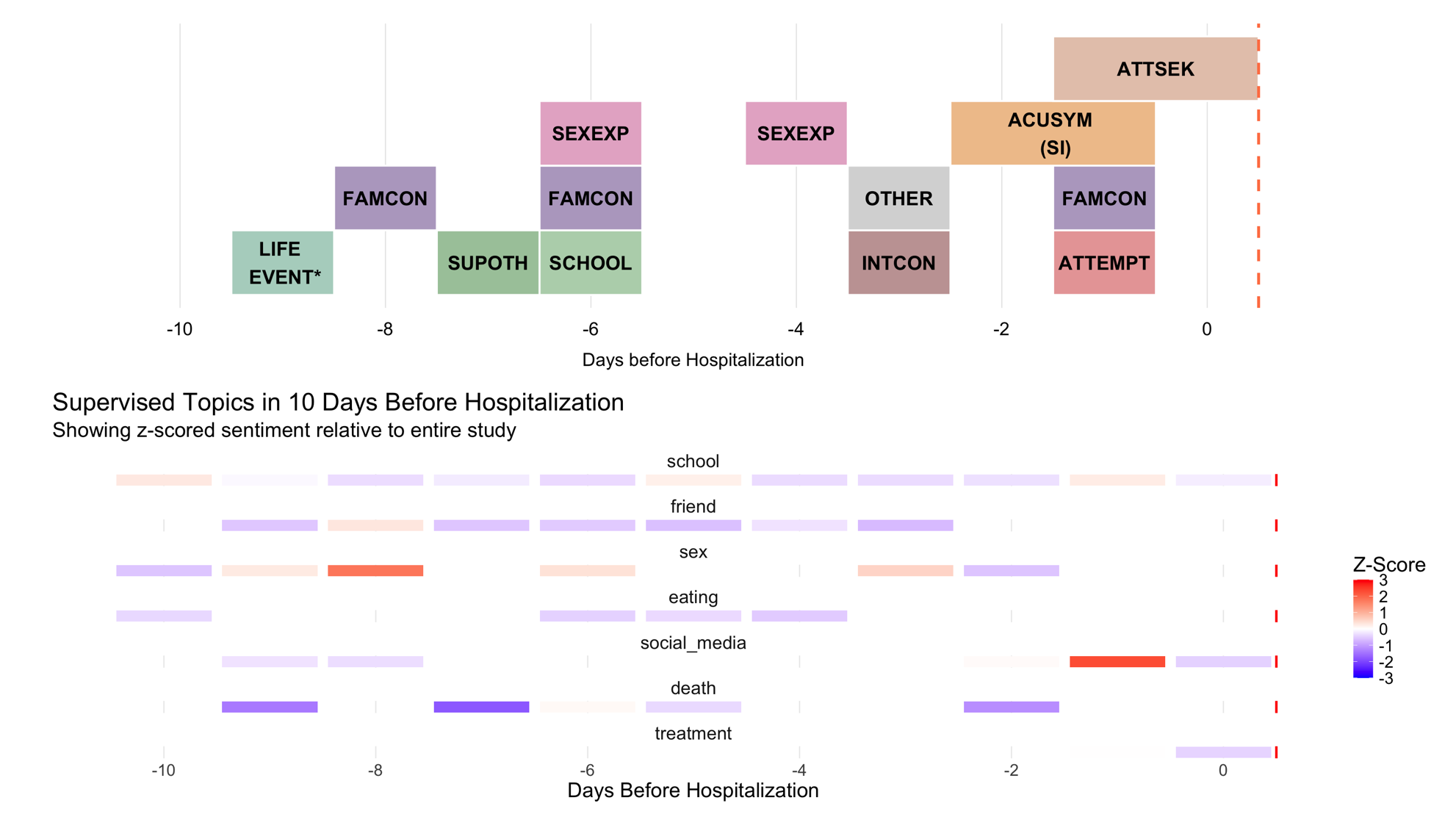
Figure S16: Timeline and Topic Sentiment Correspondence Plot, Case 3.


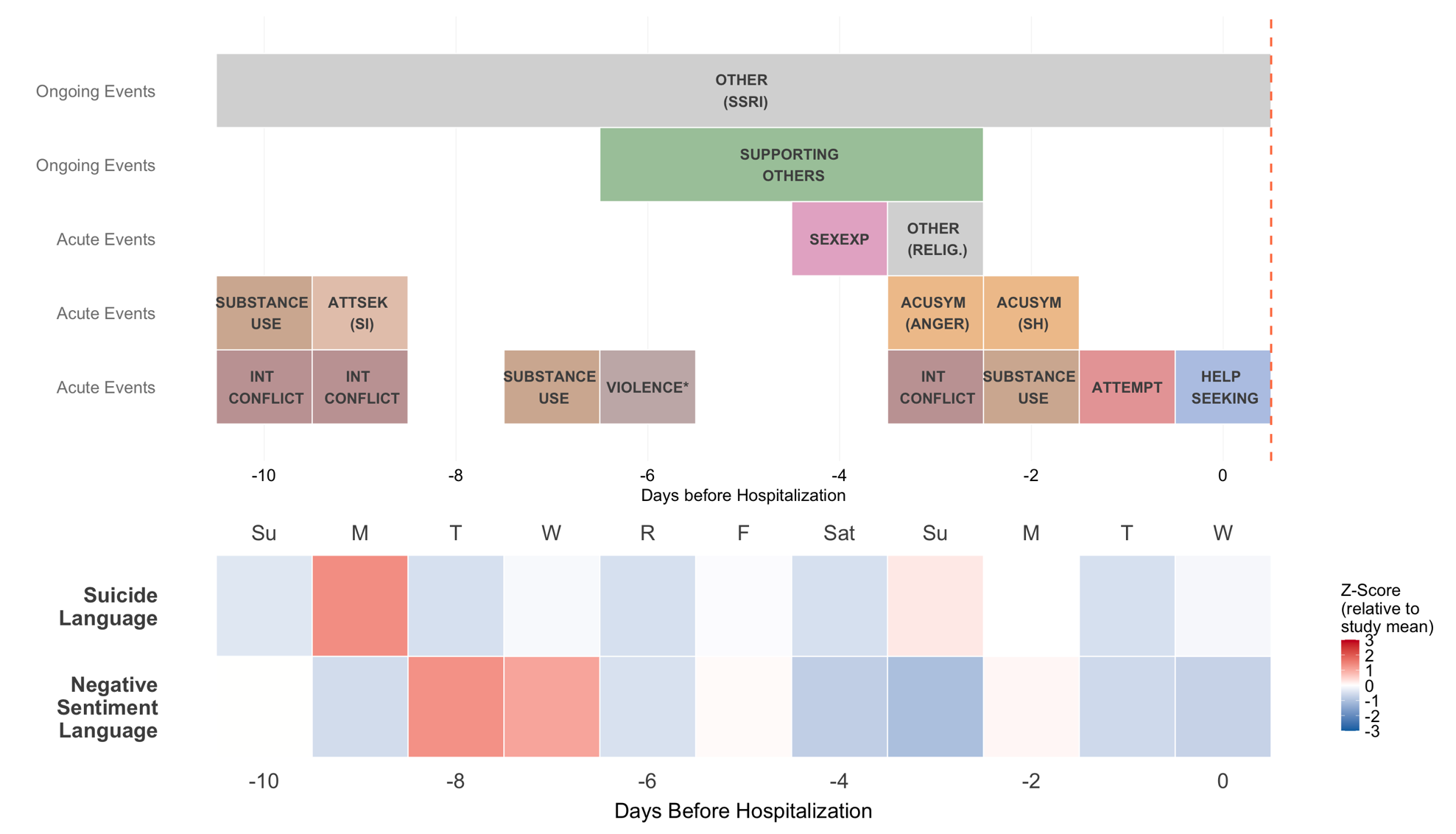


Figure S17: Timeline, Sentiment and Suicidal Language Correspondence Plot, Case 4. VIOLEN*: reflects threatening online language.

##
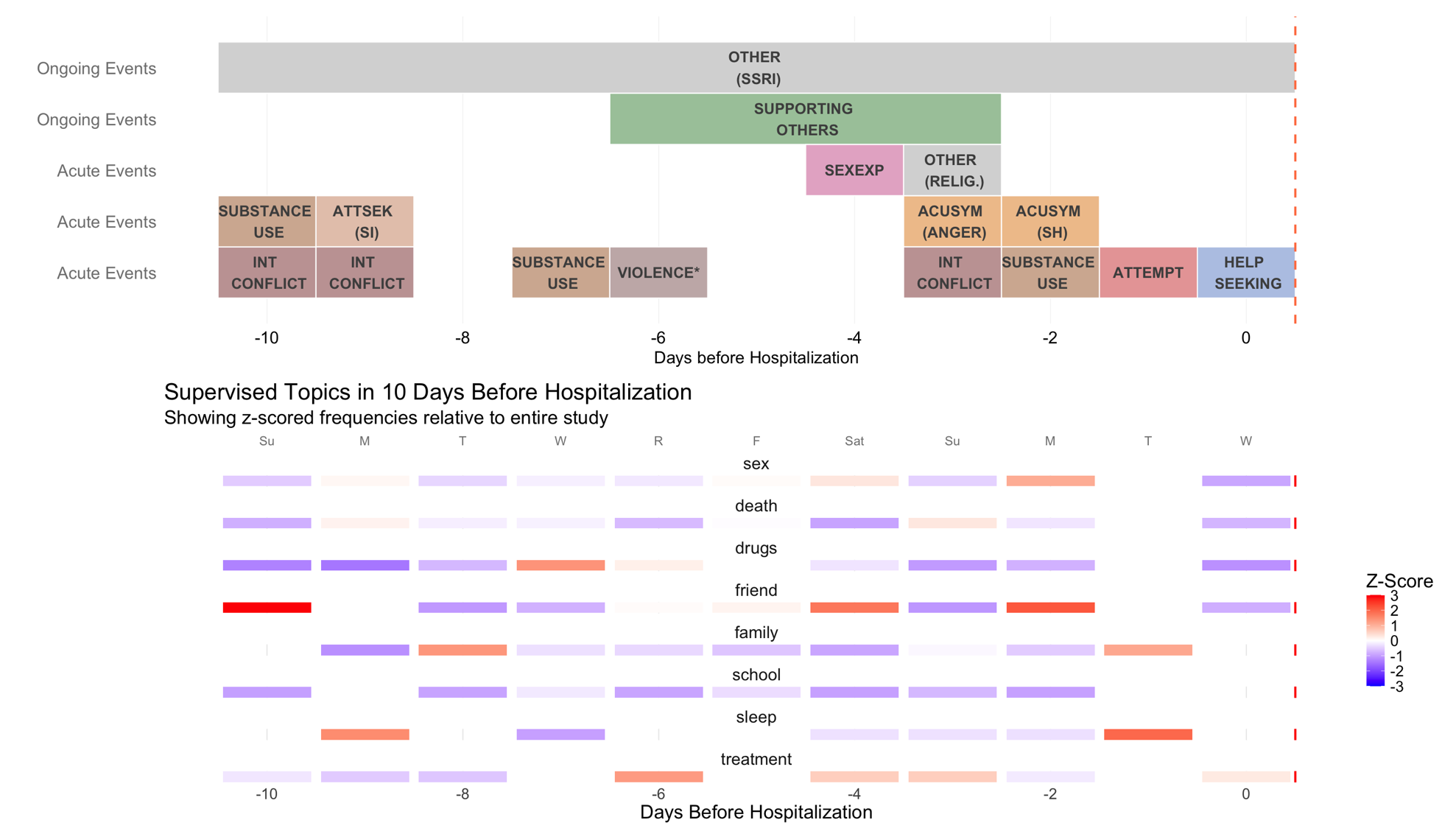
 Figure S17: Timeline and Topic Frequency Correspondence Plot, Case 4.


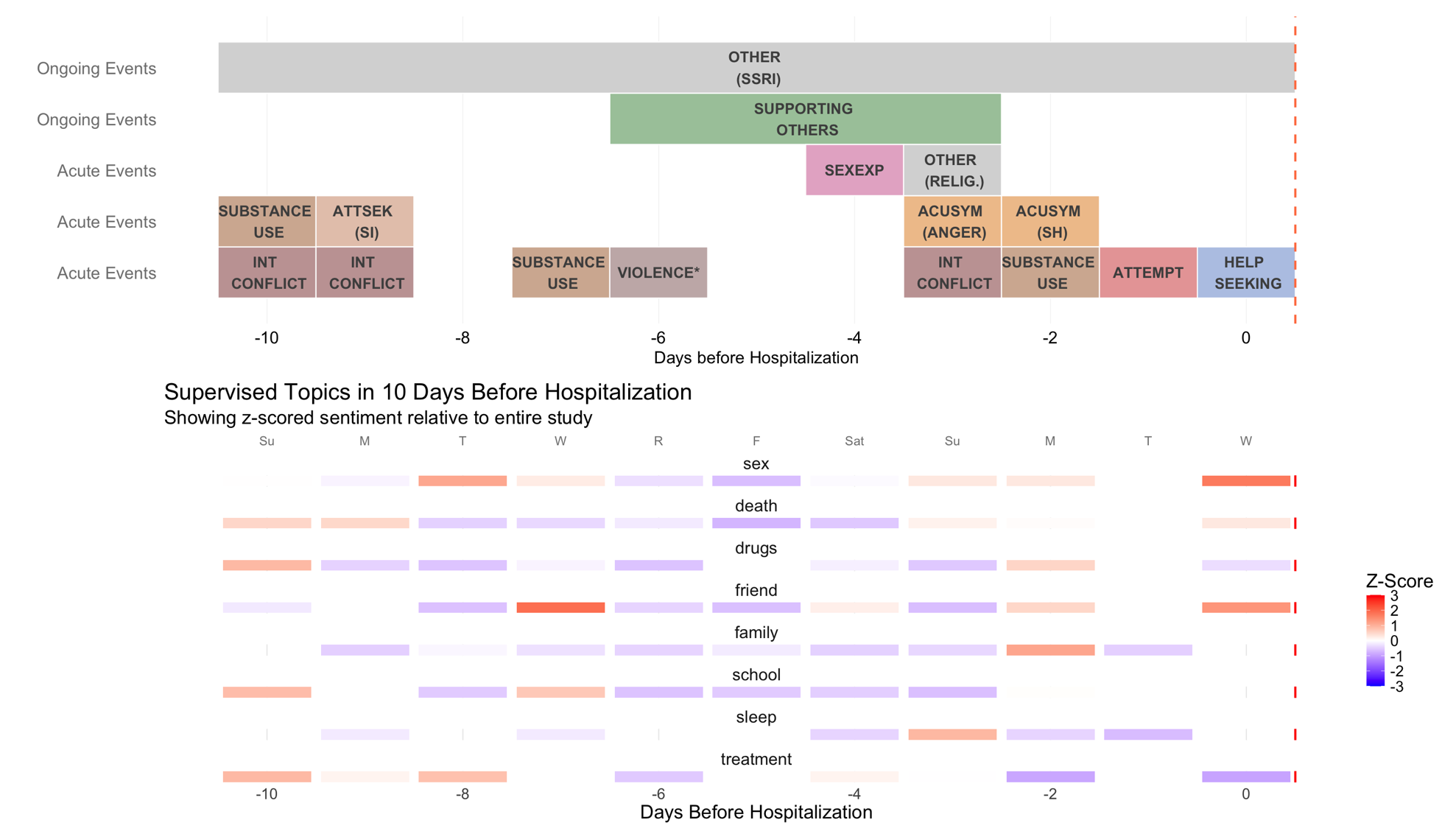


## Figure S18: Timeline and Topic Sentiment Correspondence Plot, Case 4.

##
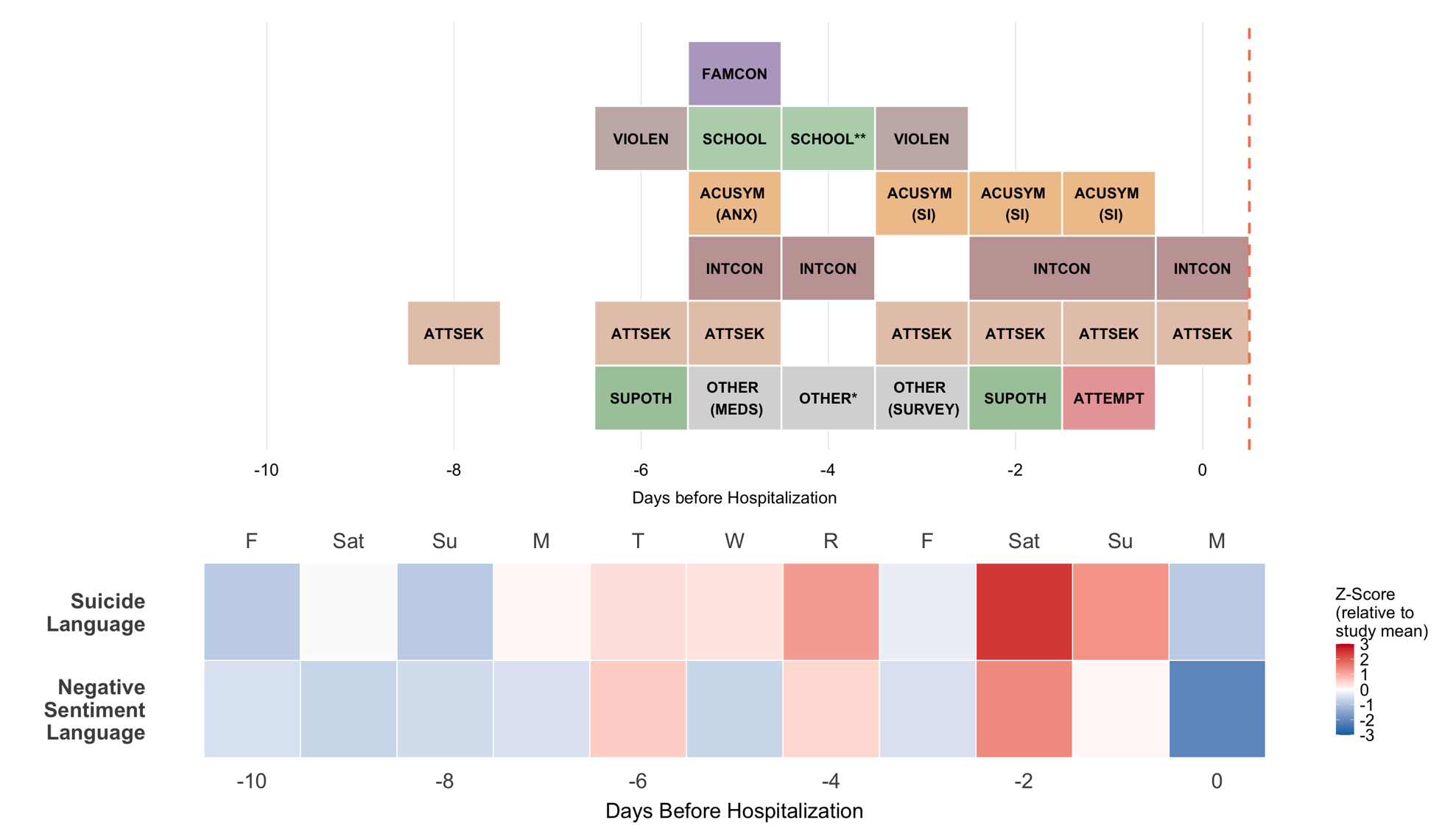
Figure S19: Timeline, Sentiment and Suicidal Language Correspondence Plot, Case 5.

OTHER (MEDS): reflects forgetting to take meds. OTHER (SURVEY): reflects taking a survey about STB. OTHER*: therapy session. SCHOOL**: academic success.

##
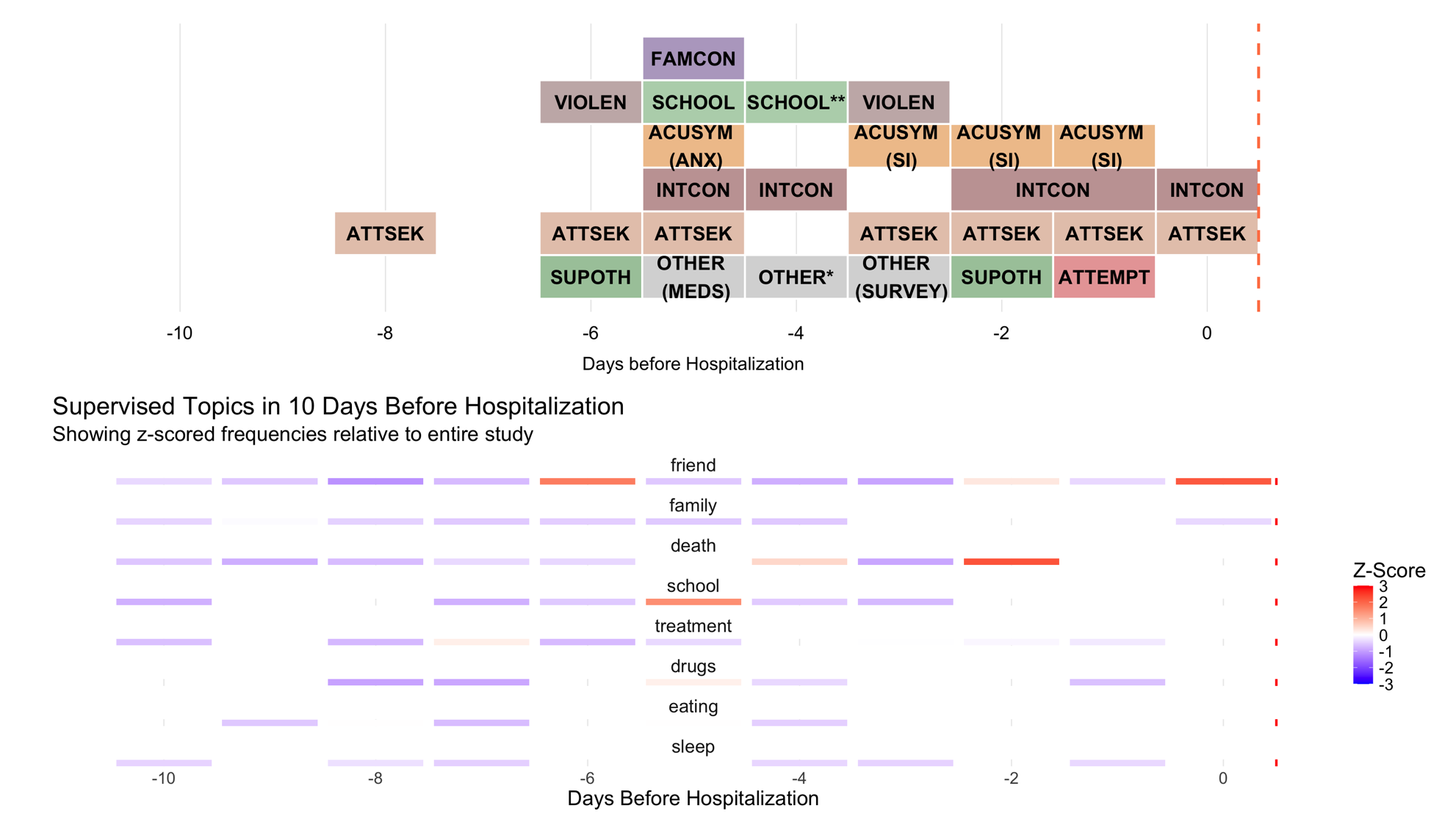
Figure S20: Timeline and Topic Frequency Correspondence Plot, Case 5.


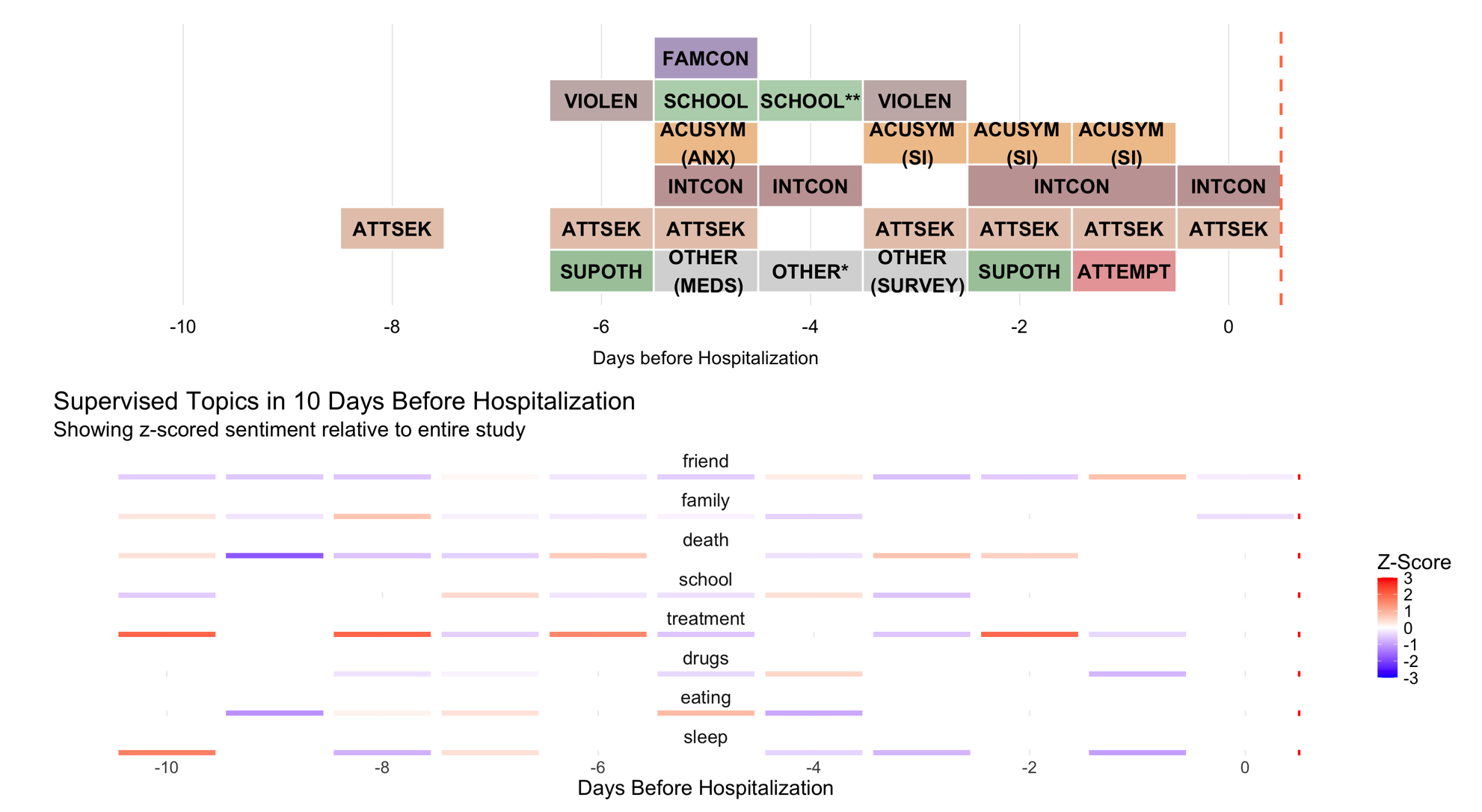
Figure S21: Timeline and Topic Sentiment Correspondence Plot, Case 5.


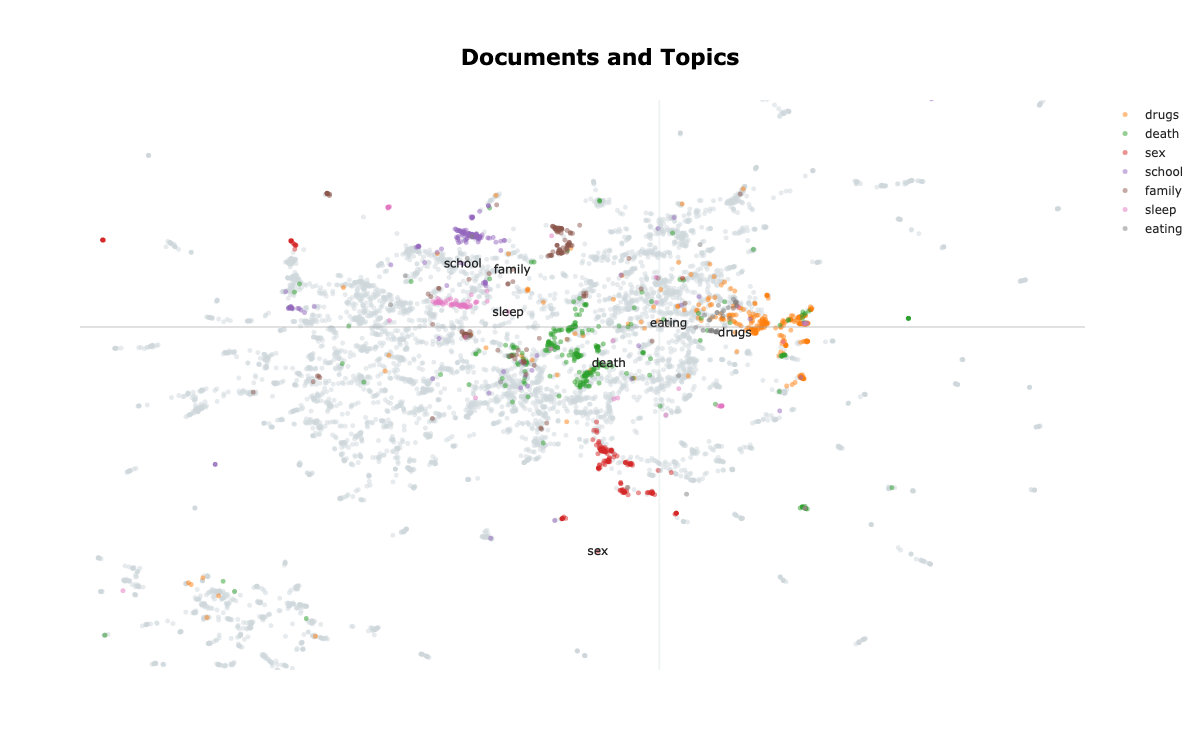


Figure S22: Embeddings for Case 1. It may be observed that sleep (pink), along with family (brown), are widely dispersed. Sex, drugs (substance use), school, and death are more clustered.

# References

1. Sheehan, D. V. *et al.* The Mini-International Neuropsychiatric Interview (MINI): the development and validation of a structured diagnostic psychiatric interview for DSM-IV and ICD-10. *J. Clin. Psychiatry* **59**, 22–33 (1998).

2. Nock, M. K., Holmberg, E. B., Photos, V. I. & Michel, B. D. Self-Injurious Thoughts and Behaviors Interview: development, reliability, and validity in an adolescent sample. (2007).

3. Posner, K. *et al.* The Columbia–Suicide Severity Rating Scale: Initial Validity and Internal Consistency Findings From Three Multisite Studies With Adolescents and Adults. *Am. J. Psychiatry* **168**, 1266–1277 (2011).

4. Su, C. *et al.* Machine learning for suicide risk prediction in children and adolescents with electronic health records. *Transl. Psychiatry* **10**, 1–10 (2020).

5. Franklin, J. C. *et al.* Risk factors for suicidal thoughts and behaviors: A meta-analysis of 50 years of research. *Psychol. Bull.* **143**, 187 (2017).

6. Camacho-Collados, J. *et al.* TweetNLP: Cutting-edge natural language processing for social media. *ArXiv Prepr. ArXiv220614774* (2022).

7. Antypas, D., Preece, A. & Camacho-Collados, J. Negativity spreads faster: A large-scale multilingual twitter analysis on the role of sentiment in political communication. *Online Soc. Netw. Media* **33**, 100242 (2023).

8. Treves, I. N. *et al.* Repetitive Negative Thinking and Adolescent Suicide: Transformer-based Approaches in Natural Language Processing. in *Decoding Naturalistic Thoughts and Behaviors with AI: Advancing Suicide Risk Detection and Clinical Applications* (2025).
